# RIP1-dependent linear and nonlinear recruitments of caspase-8 and RIP3 respectively to necrosome specify distinct cell death outcomes

**DOI:** 10.1007/s13238-020-00810-x

**Published:** 2021-01-02

**Authors:** Xiang Li, Chuan-Qi Zhong, Rui Wu, Xiaozheng Xu, Zhang-Hua Yang, Shaowei Cai, Xiurong Wu, Xin Chen, Zhiyong Yin, Qingzu He, Dianjie Li, Fei Xu, Yihua Yan, Hong Qi, Changchuan Xie, Jianwei Shuai, Jiahuai Han

**Affiliations:** 1 State Key Laboratory of Cellular Stress Biology, School of Life Sciences, Innovation Center for Cell Signaling Network, Xiamen University, Xiamen, 361102 China; 2 Department of Physics, Xiamen University, Xiamen, 361005 China; 3 National Institute for Data Science in Health and Medicine, Xiamen, 361102 China; 4 Complex Systems Research Center, Shanxi University, Taiyuan, 030006 China; 5 School of Medicine, Cancer Research Center of Xiamen University, Xiamen, 361102 China

**Keywords:** necrosome, protein complexes quantification, RIP1, SWATH-MS, network modeling

## Abstract

There remains a significant gap in our quantitative understanding of crosstalk between apoptosis and necroptosis pathways. By employing the SWATH-MS technique, we quantified absolute amounts of up to thousands of proteins in dynamic assembling/de-assembling of TNF signaling complexes. Combining SWATH-MS-based network modeling and experimental validation, we found that when RIP1 level is below ~1000 molecules/cell (mpc), the cell solely undergoes TRADD-dependent apoptosis. When RIP1 is above ~1000 mpc, pro-caspase-8 and RIP3 are recruited to necrosome respectively with linear and nonlinear dependence on RIP1 amount, which well explains the co-occurrence of apoptosis and necroptosis and the paradoxical observations that RIP1 is required for necroptosis but its increase down-regulates necroptosis. Higher amount of RIP1 (>~46,000 mpc) suppresses apoptosis, leading to necroptosis alone. The relation between RIP1 level and occurrence of necroptosis or total cell death is biphasic. Our study provides a resource for encoding the complexity of TNF signaling and a quantitative picture how distinct dynamic interplay among proteins function as basis sets in signaling complexes, enabling RIP1 to play diverse roles in governing cell fate decisions.

**Electronic supplementary material:**

The online version of this article (10.1007/s13238-020-00810-x) contains supplementary material, which is available to authorized users.

## Introduction

Tumor necrosis factor (TNF) can induce apoptosis or necroptosis depending on cellular contexts, and apoptosis and necroptosis pathways can compete with and convert into each other (Han et al., 2011; Brenner et al., 2015). TNF signaling is initiated by TNF-TNFR1 (TNF receptor 1) ligation that promotes the formation of complex I. Complex I mainly consists of TNFR1-associated death domain protein (TRADD), receptor-interacting protein kinase 1 (RIP1), TNF receptor-associated factor 2 (TRAF2), cellular inhibitor of apoptosis 1/2 (cIAP1/2) and some other proteins. Polyubiquitination of RIP1 occurs in complex I, leading to NF-κB activation. The non-ubiquitinated or de-ubiquitinated RIP1 recruits Fas-associated death domain protein (FADD) and caspase-8 to form complex II, which allows caspase-8 auto-processing, resulting in apoptosis (Brenner et al., 2015). If there is RIP3 expression in cells, RIP3 is recruited to the RIP1-FADD-caspase-8 complex to form necrosome. Autophosphorylation of RIP1 and RIP3 occurs in necrosome and phosphorylated RIP3 recruits mixed lineage kinase domain-like protein (MLKL) (Zhang et al., 2009; Cai et al., 2014), which is then phosphorylated by RIP3 and released from necrosome to execute necroptosis. Caspase-8 exhibits low level protease activity in necrosome which is responsible for the cleavage and inactivation of RIP1 and RIP3 to halt necroptotic process (Tummers et al., 2017; Newton et al., 2019a; Newton et al., 2019b).

RIP1 is a key molecule in diverting TNF-induced NF-κB activation, apoptosis and necroptosis (Ofengeim and Yuan, 2013). Despite the requirement of RIP1 in cell death, cell-based experiments uncovered that RIP1 suppresses RIP3/MLKL-mediated necroptosis (Orozco et al., 2014). The inhibitory function of RIP1 on cell death was also demonstrated in mouse genetic studies (Dillon et al., 2014; Rickard et al., 2014; Kaiser et al., 2014; Newton et al., 2016). *Rip1*^−/−^ mice were perinatally lethal and exhibited apoptosis in multiple tissues (Rickard et al., 2014). Gene deletion of caspase-8 cannot rescue *Rip1*^−/−^ mice but eliminated apoptosis (Rickard et al., 2014), suggesting that RIP1 deficiency unleashes caspase-8 activity. Deletion of RIP3 also had minimal effect on the survival of *Rip1*^−/−^ mice, but *Rip1*^−/−^*Rip3*^−/−^*Casp8*^−/−^ mice survived until adulthood (Dillon et al., 2014; Rickard et al., 2014). Further genetic analysis suggested that RIP1 inhibits TNF-induced FADD/caspase-8-dependent apoptosis and TRIF/IFN-mediated RIP3/MLKL-dependent necroptosis (Dillon et al., 2014; Kaiser et al., 2014). The follow-up study pinpointed that ZBP1, but not TRIF/IFN, is essential for RIP3/MLKL-dependent necroptosis caused by RIP1 deletion (Newton et al., 2016). Therefore, RIP1 can either promote or limit necroptosis under certain conditions (Weinlich and Green, 2014). But the mechanism underlying this paradoxical role of RIP1 has not been elucidated.

Protein interactions are essential in determining signaling consequences. Quantification study of these interactions could clarify how death signals are integrated to direct a specific cell fate. Combination of modeling and experimental approaches has been used to reveal essential features and control circuits of various cellular signaling pathways (Ma et al., 2009; Nakakuki et al., 2010; Yang et al., 2018; Bashor et al., 2019), including NF-κB and apoptosis pathways (Albeck et al., 2008; Shinohara et al., 2014). However, achieving quantitative mechanistic insights is still a major challenge because of limited consistent and comprehensive data in experiments (Kitano, 2005; Cox and Mann, 2011). Recent advances in the quantification capability of proteomics are now able to overcome this limitation (Aebersold and Mann, 2016). To quantitatively analyze the dynamic formation of TNF-induced signaling complexes of cell death, we employed immune-purification coupled with proteomics. SWATH-MS (sequential window acquisition of all theoretical fragment ion spectra mass spectrometry) technique (Ludwig et al., 2018) was used to obtain reproducible and absolute protein amounts across multiple samples, which are currently the most sensitive inputs for mathematical modeling. Using the SWATH-MS-based modeling and experimental analysis, our study provides a quantitative framework for understanding how RIP1 distinctly recruits RIP3 and pro-caspase-8 into necrosome, thereby determining cell death type through influencing necrosome assembling and de-assembling, and also shows a biphasic relationship between RIP1 level and the occurrence of necroptosis or the total cell death.

## Results

### Absolute quantification of proteins in TNF signaling complexes by SWATH-MS

**Figure 1:**
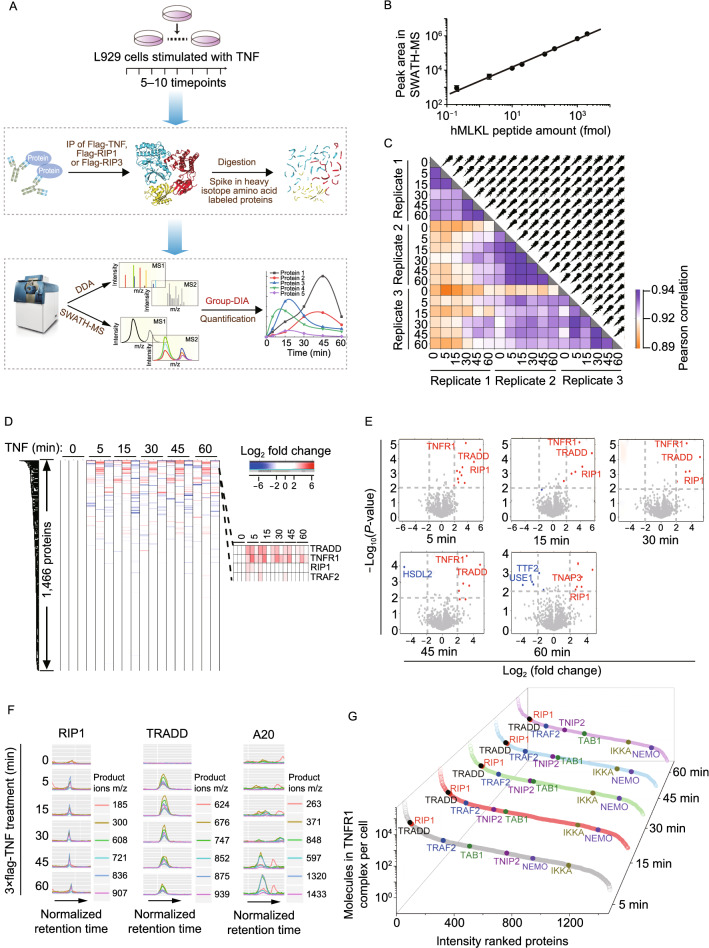
**Application of IP-SWATH workflow in quantifying TNF signaling complexes.** (A) Experimental scheme of IP-SWATH workflow. L929 cells were treated with Flag-TNF or TNF (10 ng/mL) for 5 to 10 timepoints, and TNFR1, RIP1 and RIP3 complexes were immunoprecipitated with anti-Flag agarose beads. Purified heavy isotope amino acid-labeled targeted proteins were spiked into the IP samples. IP samples were digested with trypsin and peptides were analyzed using DDA and SWATH-MS. Group-DIA was employed to analyze SWATH-MS data for generation of pseudo-spectra files. DDA files and pseudo-spectra files were subjected to database searches, followed by targeted analysis of SWATH-MS. (B) Dynamic range of SWATH-MS. Different amounts of purified recombinant human MLKL protein were spiked into murine MLKL knockout (KO) cell lysates in triplicates, followed by trypsin digestion. The peptide samples were analyzed using SWATH-MS. SWATH-MS data were analyzed using Peakview software, and the product ion peak area of the MLKL-specific peptide LGQLIYEQCEK was directly taken from the Peakview. The sum of top three peak areas of product ions represented the peptide amount. (C) Reproducibility analysis of quantitative protein intensities in TNFR1 dataset. The protein intensities in two samples, each contains three replicates, were compared in TNFR1 dataset, and Pearson correlations were calculated. (D) Heat map of quantitative proteins in TNFR1 dataset. The known TNFR1 complex components were indicated in the right panel. (E) Differential expression analysis of proteins at each timepoint in TNFR1 dataset. Proteins with |Log2(fold change)| > 2 and −Log10(*P*-value) > 2 were considered significantly changed. Up-regulated proteins were labeled in red, and down-regulated proteins were labeled in blue. (F) XICs of peptides of high-confidence interaction proteins in TNFR1 dataset. Traces in different colors represent different product ions of given peptides. (G) Dynamic range of all identified proteins estimated by molecules per cell (mpc) in TNFR1 dataset. Some representative interaction proteins of TNFR1 were highlighted

We employed MS to systematically analyze the dynamic assembly of three critical complexes—TNFR1, RIP1 and RIP3 complexes of TNF signaling in L929 cell line, which is a well-established cellular model to study necroptosis. The responses of wildtype, Flag-RIP1, and Flag-RIP3 cell lines to TNF stimulation were similar in terms of activation of NF-κB, MAPKs, and MLKL (Fig. S1A). For absolute protein quantification, heavy amino acid-labeled proteins were spiked into purified IP complexes, which were subsequently subjected to DDA and SWATH-MS analyses (Fig. 1A). Targeted extraction of human MLKL peptides (Fig. 1B) and β-galactosidase (Fig. S1B**)** using Peakview software from SWATH-MS data revealed that excellent quantification accuracy with about 4 orders of dynamic range could be achieved (Table S1). We combined the internal library generated by Group-DIA (Li et al., 2015) and the external library built from DDA for peptide detection (Fig. S1C and Table S2). Collectively, 1,466, 1,297 and 2,355 proteins were quantified across 18, 30 and 15 IP samples in TNFR1, RIP1 and RIP3 datasets, respectively (Table S2). Protein abundance comparison showed that Pearson correlation coefficients were about 0.89–0.94, 0.81–0.94, and 0.87–0.96 between any two different runs for TNFR1, RIP1 and RIP3 datasets respectively (Figs. 1C and S1D). Coefficients of variation of log2-transformed protein abundance at all time-points were below 10% (Fig. S1E). Thus, the entire experiment is excellent in reproducibility.

Hierarchical clustering of TNFR1, RIP1 and RIP3 interaction proteins indicated a small number of proteins changed in abundance after TNF treatment (Figs. 1D and S1F), suggesting a high level of non-specific background proteins. We therefore distinguished specifically recruited proteins from the background proteins by analyzing the amount change of proteins in complexes at different time periods. Differential expression analysis of each individual protein at different time points was conducted and shown in Figs. 1E and S1G. Manual inspection of all XICs (extracted ion chromatograms) of significantly changed proteins in Figs. 1E and S1G indicated that 11, 9 and 3 proteins were recruited to TNFR1, RIP1 and RIP3 complexes, respectively (Table S2). The representative peptide XICs of RIP1, TRADD and A20 of the 11 proteins recruited to TNFR1 complex were shown in Fig. 1F. These identified proteins are components of the complexes (Brenner et al., 2015), confirming the accuracy of our identification by IP-SWATH.

We quantified absolute amounts of key proteins in TNFR1, RIP1 and RIP3 complexes by using the spiked-in purified heavy amino acids-labeled proteins. The amounts of proteins in the complexes were then estimated by the “TOP3 peptides” approach (Table S3) (Al Shweiki et al., 2017). Absolute amounts of proteins in these three complexes are shown in Figs. 1G and S1H. The significantly changed proteins in TNFR1 complex are highlighted (Fig. 1G). Collectively, our IP-SWATH workflow provides an efficient method to accurately identify and absolutely quantify the dynamically assembled proteins in TNF signaling complexes.

### SWATH-MS-based modeling of TNF-induced cell death signaling network

**Figure 2:**
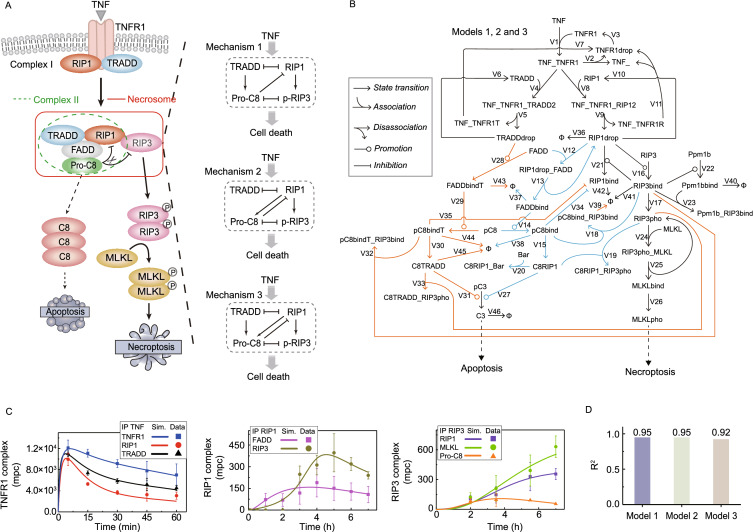
**SWATH-MS-based modeling of TNF-induced cell death signaling pathway.** (A) Diagram of TNF-induced complexes assembly and components relation. The circled letter P denotes phosphorylation state. Simplified schematic diagrams that represent three different regulation mechanisms of pro-caspase-8 (Pro-C8) by TRADD (Mechanism 1), RIP1 (Mechanism 2) or TRADD and RIP1 (Mechanism 3) are shown in the right panel. Mutual inhibition between TRADD and RIP1 is presented to describe the competitive relation between the two proteins binding TNFR1. (B) Kinetic scheme of Models 1, 2 and 3. Lines ended by symbols represent the chemical reactions characterized by Reactions V1-V44 in Table S4. Parameters for the individually numbered reactions are given in Table S6. Ø represents degraded proteins. Model 1 (Mechanism 1) includes the reactions described by black and orange lines, whereas Model 2 (Mechanism 2) involves the reactions described by black and blue lines. Model 3 (Mechanism 3) contains all reactions. (C) Simulation results using Model 1 (lines) and SWATH-MS data (dots) of the time-course responses upon TNF stimulation. Error bars denote standard deviation (SD) for three independent experiments. The unit is molecules per cell (mpc). (D) Deviation (R^2^) between simulation results of the three models and SWATH-MS data

The amounts of the 11, 9 and 3 proteins recruited to TNFR1, RIP1 and RIP3 complexes respectively ranged from ~20 to 12,000 mpc as determined by our SWATH-MS (Table S3). The high-abundant proteins are likely to be signaling transducers whereas the others could be catalysts. We therefore simplified illustration of TNF-induced signaling pathway by using these abundant proteins as transducers (Fig. 2A). Complex I is formed by TRADD and RIP1, competitively binding to TNFR1. Complex II is composed of RIP1, FADD and caspase-8, which has the same components as necrosome except for lacking RIP3. In necrosome, activated caspase-8 cleaves and inactivates RIP1 and RIP3 to inhibit necroptosis. Uncleaved RIP3 phosphorylates and recruits MLKL, eventually leading to necroptosis (Han et al., 2011; Berghe et al., 2014). Experimental data suggest that pro-caspase-8 can be either activated by TRADD (Mechanism 1) or RIP1 (Mechanism 2) independently or by TRADD and RIP1 together (Mechanism 3) (Fig. 2A, right panel) (Vanlangenakker et al., 2011; Duprez et al., 2012; Berghe et al., 2014; Remijsen et al., 2014). Accordingly, we developed three corresponding mathematical models, Model 1 with Mechanism 1, Model 2 with Mechanism 2 and Model 3 with Mechanism 3, which incorporated protein interactions, phosphorylation, cleavage and enzymatic reactions (Fig. 2B). By comparing the dynamics of these models to experimental data, we can quantitatively explore the emergent properties of TNF signaling complexes. All the biochemical reactions in Fig. 2B (V1-V46) and reaction rates in models are elaborated in Table S4. These models are described by a cast of ordinary differential equations (ODEs), which are represented by compounds and kinetic parameters based on the law of mass action. Complete model descriptions are presented in the Supplementary Materials. To examine the reliability of the models, simulation curves were compared with the SWATH-MS data shown in Figs. 1G and S1H. Comparison results indicated that Models 1, 2 and 3 can well reproduce the amounts of major components in TNFR1, RIP1 and RIP3 complexes obtained by SWATH-MS (Figs. 2C and S2). Squares of the correlation coefficients (R^2^) between SWATH-MS data and simulation results are all larger than 0.9 for these three models (Fig. 2D). Hence, all of these models exhibit high confidence for further clarifying the transduction mechanism how RIP1 is formed in complexes to exert strategic control, thereby governing cell fate.

### Biphasic roles of RIP1 in RIP3-dependent necroptosis

**Figure 3:**
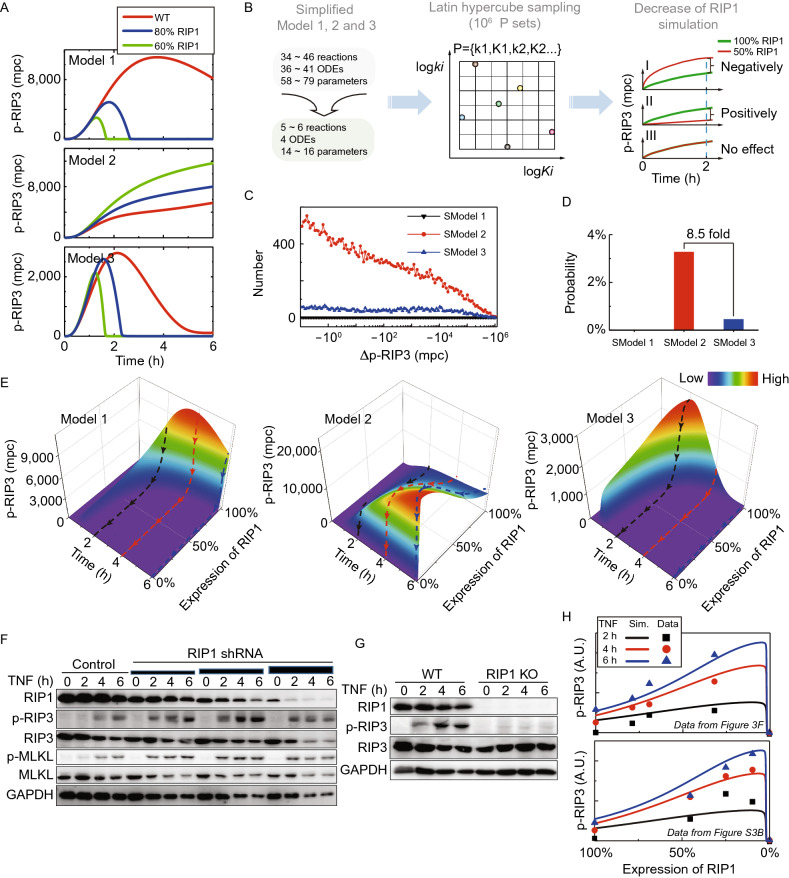
**Roles of RIP1 in RIP3 phosphorylation mediated necroptosis.** (A) Simulated effects of three different RIP1 expression levels on p-RIP3 in Models 1, 2 and 3 upon TNF stimulation. (B) Illustration of the analyzing procedure for randomly parameterized models. (C) Parameter set number distributions of Δp-RIP3 < 0 obtained by randomly parameterized simplified models. (D) Probability of Δp-RIP3 < 0 deduced from simplified models. (E) Simulated effects of various degrees of RIP1 decrease on p-RIP3 in Models 1, 2 and 3. p-RIP3 changes mediated by RIP1 decrease after 2, 4 and 6 h TNF treatment are respectively shown with black, red and blue dashed lines. (F) RIP1 was knocked down (KD) to different levels in cells. The cells were then analyzed by Western blot with the indicated antibodies. (G) Wildtype (WT) and RIP1 knockout (KO) cells were treated with TNF and then were analyzed by Western blot. (H) Comparison between Model 2 predictions and experimental data of p-RIP3 regulated by RIP1 level. Quantified experimental data obtained with Western blot in (F) and replicates (Fig. S3B) are shown in upper and down panels. RIP3 phosphorylation data were normalized to the corresponding expression level of the total RIP3. A.U. denotes the arbitrary unit

We next applied these three models to quantitatively dissect the functional roles of RIP1 in necroptosis. RIP3 phosphorylation is used as the marker of necroptosis (Zhang et al., 2009). RIP1 level in wildtype cells is treated as 100% RIP1, which is ~51,000 ± 3,100 mpc based on three independent SWATH-MS data. By simulation, the reduction of RIP3 phosphorylation is associated with a decrease of RIP1 level in both Model 1 and Model 3 (Fig. 3A, top and bottom panels), but with an increase of RIP1 level in Model 2 (Fig. 3A, middle panel). Only Model 2 shows a negative regulation of RIP3 phosphorylation by the increase of RIP1 level.

To avoid bias, we also used random parameters instead of SWATH-MS data-optimized parameters in these models for analysis. As each of Models 1, 2 and 3 contains more than 50 parameters (Table S6), we reduced parameters to 14, 14 and 16 respectively for simplified Model 1 (SModel 1), Model 2 (SModel 2), and Model 3 (SModel 3) (Fig. 3B and Supplementary Materials) to make the simulation feasible. Random parameter sets were generated through a Monte Carlo approach. For each SModel, we sampled 10^6^ random parameter sets using Latin hypercube sampling method and then for each parameter set determined how RIP1 regulates RIP3 phosphorylation. The positive or negative regulation can be evaluated by the value of Δp-RIP3, which is the level difference of RIP3 phosphorylation between wildtype and the cell containing 50% RIP1 (Fig. 3B). The number of parameter sets that yield negative regulation of RIP3 phosphorylation by RIP1 was calculated for each SModel and the corresponding set number distributions are presented in Fig. 3C. The results indicated that zero parameter set occurs to generate negative regulation with SModel 1 (black line), while certain numbers of parameter sets are obtained with SModels 2 and 3. SModel 2 gives a much larger set number than SModel 3 (Fig. 3C, red and blue lines). Percentage of the summed set numbers in all sampled parameter sets which is referred to as probability, is shown in Fig. 3D. Thus, sampling of random parameters also substantiates that, rather than SModels 1 and 3, SModel 2 is more highly preferred to robustly achieve the negative regulation of RIP3 phosphorylation by RIP1. Gillespie algorithm (Gillespie, 1977) was also performed to evaluate the negative regulation of RIP3 phosphorylation by RIP1 and the result is similar (Supplementary Materials).

To make further comprehensive analysis, RIP3 phosphorylation was simulated under a serial of RIP1 levels (Fig. 3E). As expected, RIP1 down-expression reduces RIP3 phosphorylation in Models 1 and 3, but enhances RIP3 phosphorylation only in Model 2. Model 2 predicted that RIP3 phosphorylation reaches its maximum level as RIP1 decreases to an extremely low level (~1,000 mpc, e.g., ~2% of wildtype), and then rapidly declines when RIP1 reduces further, showing a biphasic relationship of RIP3 phosphorylation to RIP1 level.

To test our model prediction, we experimentally knocked down RIP1 to three different expression levels with RIP1-specific short hairpin RNA (shRNA), and detected the increase of TNF-induced phosphorylation of RIP3 and MLKL (Fig. 3F). Similar results were obtained when another RIP1 shRNA was used (Fig. S3A). Deletion of RIP1 completely blocked TNF-induced RIP3 phosphorylation (Fig. 3G). The band intensities in Fig. 3F and 3G and a replicate (Fig. S3B) were compared with Model 2 prediction (Fig. 3H), quantitatively confirming the biphasic regulation of RIP3 phosphorylation by RIP1. Therefore, RIP1 functions as an activator in necroptosis within extremely low level range (<~2% of wildtype) and as an inhibitor at higher level.

### Linear and nonlinear recruitments of pro-caspase-8 and RIP3 to necrosome

**Figure 4:**
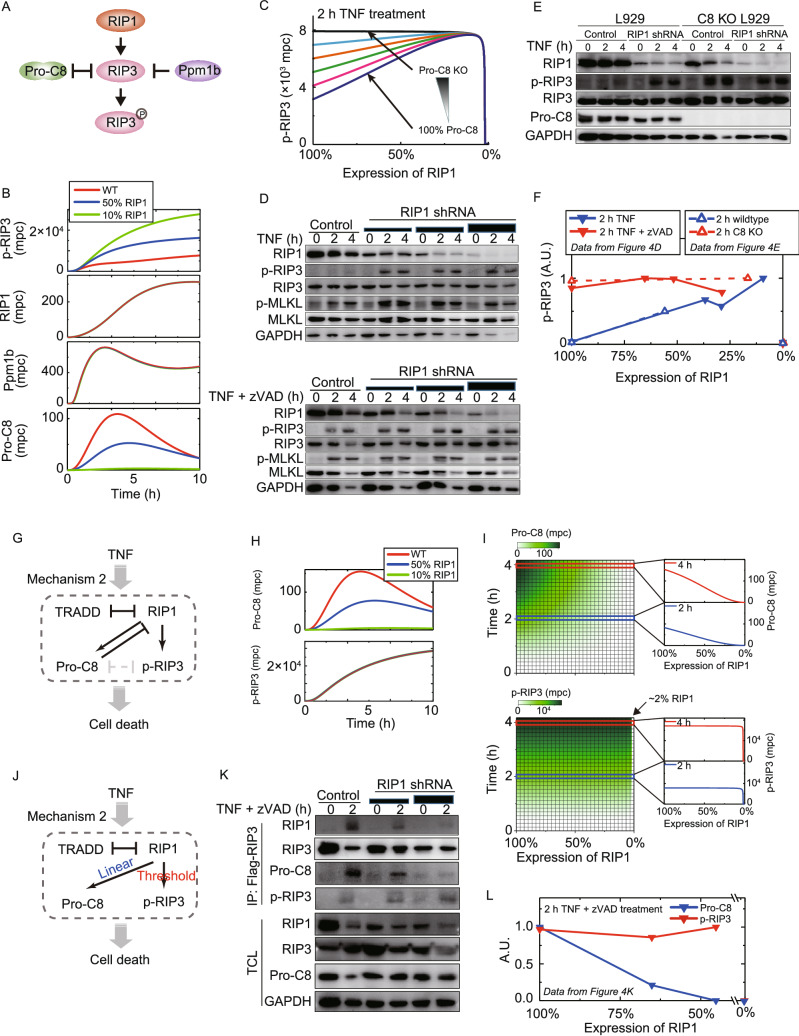
**Distinct dynamic responses of Pro-C8 and p-RIP3 to RIP1 decrease.** (A) Schematic diagram of components related to p-RIP3 in Model 2. (B) Simulated effects of RIP1 levels on RIP1, Ppm1b and Pro-C8 that interact with RIP3. (C) Simulated effects of Pro-C8 expression decrease on RIP1 KD induced increase of p-RIP3. (D) RIP1 KD cells were treated with TNF or TNF plus zVAD and then were analyzed by Western blot. (E) RIP1 was knocked down in caspase-8 KO cells. The cells were then analyzed by Western blot after TNF treatment. (F) Quantified Western blot results of p-RIP3 in (D) and (E). (G) Schematic diagram of Mechanism 2 (Model 2) after removing the inhibition between Pro-C8 and RIP3. (H) Simulated effects of RIP1 levels on Pro-C8 that binds to RIP1 and p-RIP3 after removing Pro-C8 and RIP3 interaction in Model 2. (I) Simulation of a serial of RIP1 expression levels on Pro-C8 that binds to RIP1 and p-RIP3. (J) Summary diagram: Pro-C8 exhibits linear response and p-RIP3 shows ultrasensitive threshold response to RIP1 decrease. (K) RIP1 was knocked down in Flag-RIP3 expressing RIP3 KO cells. The cells were analyzed by Western blot. (L) Quantified Western blot results in (K) of p-RIP3 and Pro-C8 in RIP3 complex

To dissect the underlying mechanism how RIP1 biphasically regulates RIP3 phosphorylation, we employed Model 2 to examine the dynamic behaviors of RIP1, pro-caspase-8, and Ppm1b (Chen et al., 2015), which are known to modulate RIP3 phosphorylation in necrosome (Fig. 4A). When RIP1 level decreased, RIP3 phosphorylation increased, and the amounts of RIP1 and Ppm1b that interacted with RIP3 were barely influenced (Fig. 4B). However, decrease of RIP1 reduced pro-caspase-8 that was associated with RIP3. Since pro-caspase-8 cleaves RIP1/RIP3 in necrosome, we introduced a reduction of pro-caspase-8 level in Model 2 (Figs. 4C and S4). Simulation results indicated that RIP3 phosphorylation exhibited a gradual increase when pro-caspase-8 level decreased. However, the trend of RIP1 decrease-associated increase of RIP3 phosphorylation became less pronounced when pro-caspase-8 level decreased. Such increase of RIP3 phosphorylation no longer occurred when pro-caspase-8 expression reduced to zero. Therefore, simulation results suggested that pro-caspase-8 activity is required for the up-regulation of RIP3 phosphorylation by decreasing RIP1 level.

We utilized z-Val-Ala-DL-Asp-fluoromethylketone (zVAD), a pan-caspase inhibitor to experimentally validate the prediction. RIP1 knockdown led to an increase of RIP3 phosphorylation upon TNF treatment (Fig. 4D, top panel). In comparison, RIP1 knockdown barely affected RIP3 phosphorylation when zVAD was included (Fig. 4D, bottom panel). In addition, decrease of RIP1, which enhanced RIP3 phosphorylation in wildtype cells, did not influence RIP3 phosphorylation in caspase-8 KO cells (Fig. 4E). The band intensities of RIP1 level and phosphorylated RIP3 in Western blot were quantified and plotted in Fig. 4F, supporting the idea that pro-caspase-8 recruitment to necrosome is essential for RIP1 negatively regulating RIP3 phosphorylation.

The suppression of RIP3 phosphorylation by pro-caspase-8 requires RIP1 in necrosome (Tummers et al., 2017). To independently address the effects of RIP1 on pro-caspase-8 activity and RIP3 phosphorylation, we made an analysis by removing the inter-inhibition between pro-caspase-8 and RIP3 in Model 2 (Fig. 4G). Simulation with three different RIP1 expression levels suggested that when RIP1 level decreased, the amount of pro-caspase-8 binding to RIP1 was reduced, but RIP3 phosphorylation kept constant (Fig. 4H). Further comprehensive simulations predicted that decrease of RIP1 results in a progressive decrease of pro-caspase-8 recruitment to necrosome, whereas RIP3 phosphorylation is barely influenced by decreasing RIP1 level from 100% to 2% and then presents a steep reduction (Fig. 4I). Thus, the response of pro-caspase-8 to RIP1 level is mostly linear, whereas RIP3 phosphorylation is not, and presents an ultrasensitive threshold pattern (Fig. 4J).

We next validated the distinct dynamic responses shown in Fig. 4J. Flag-RIP3-expressing RIP3 KO cells pre-treated with RIP1 shRNA were used to examine the behaviors of pro-caspase-8 recruitment and RIP3 phosphorylation in the cells with different RIP1 levels (Fig. 4K). The results suggested that pro-caspase-8 amounts decreased linearly in RIP3 complex when RIP1 decreased while RIP3 phosphorylation almost kept unchanged. The quantified Western blot results shown in Fig. 4L are in good agreement with the predictions in Fig. 4I, confirming the distinct dependence of pro-caspase-8 recruitment to necrosome and RIP3 phosphorylation on RIP1 level.

### RIP1-suppressed caspase-8 activation does not cleave RIP1/RIP3

**Figure 5:**
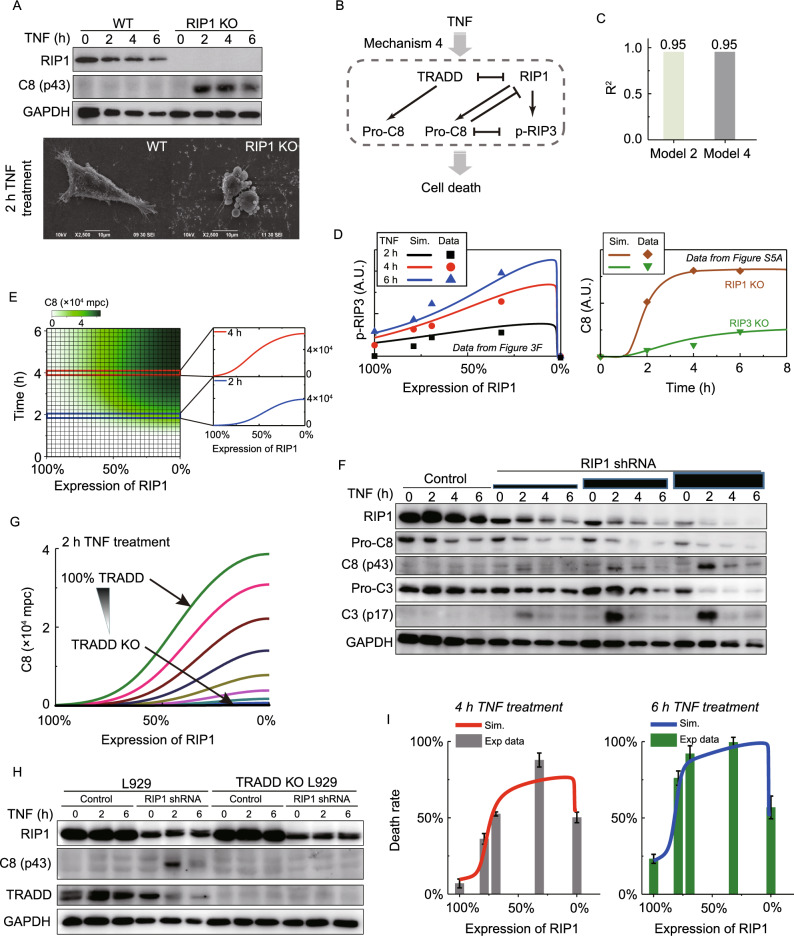
**TRADD-dependent caspase-8 activation does not cleave RIP1/RIP3.** (A) WT and RIP1 KO cells were treated with TNF and then were analyzed by Western blot. The 2 h treated cells were imaged by scanning electronic microscopy (SEM). C8 (p43): p43 fragment of caspase-8. (B) Schematic overview of Mechanism 4 in Model 4. (C) Deviation (R^2^) between simulation results of Model 4 and SWATH-MS data. (D) Comparison between Model 4 predictions and experimental data of p-RIP3 regulated by RIP1 level (left panel), C8 activation in RIP1 KO and RIP3 KO cells (right panel). Western blot data can be found in Fig. S5A. (E) Simulation of RIP1 decrease induced increase of C8 upon TNF treatment. (F) RIP1 KD cells were treated with TNF and then were analyzed by Western blot. Pro-C3: pro-caspase-3; C3 (p17): p17 fragment of caspase-3. (G) Simulated effects of TRADD reduction on RIP1 decrease induced increase of C8. (H) RIP1 was KD in WT and TRADD KO cells. The cells were then treated with TNF and were analyzed by Western blot. (I) Comparison between Model 4 predictions and experimental data of RIP1 level mediated cell death rate at 4 h and 6 h TNF treatment. Expression levels of RIP1 KD cells were quantified from Fig. 3F

Model 2 well explains RIP1’s biphasic roles in necroptosis. However, its manifestation that deletion of RIP1 can completely abolish the involvement of pro-caspase-8 conflicts with the experimental result that TNF induces quick caspase-8 activation and apoptosis in RIP1 KO cells (Fig. 5A) (Vanlangenakker et al., 2011). It is known that in Complex I, TRADD and RIP1 compete for TNFR1 binding and mediate downstream caspase-8 activation (Zheng et al., 2006; Wang et al., 2008). We therefore refined Model 2 by including additional TRADD-dependent caspase-8 activation and named it as Model 4 (Mechanism 4) (Fig. 5B). After the parameter fitting, Model 4 can reproduce not only the MS data with an R^2^ value equal to that of Model 2 (Fig. 5C) but also RIP1’s biphasic roles in necroptosis (Fig. 5D, left panel). Importantly, Model 4, but not Model 2, reveals that the activation level of caspase-8 in RIP1 KO cells is higher than that in RIP3 KO cells (right panel in Figs. 5D and S5A), which is consistent with previous studies (Vanlangenakker et al., 2011; Remijsen et al., 2014).

We next sought to dissect the function of RIP1 in apoptosis with the refined model. Simulation demonstrated that the progressive decrease of RIP1 leads to a gradual increase of caspase-8 activation (Fig. 5E). This prediction is supported by our experiment that knocking down of RIP1 promoted caspase-8 activation (Figs. 5F and S3B). Further analysis determined the induced caspase-8 activation was TRADD-dependent. When TRADD expression level was reduced in Model 4, it indeed resulted in progressive decrease of caspase-8 activation at varying RIP1 levels (Figs. 5G and S5B). Consistently, the RIP1 knockdown-induced increase of caspase-8 activation cannot be detected in TRADD KO cells (Fig. 5H). Thus, RIP1 limits apoptosis through suppressing TRADD-dependent caspase-8 activation.

Unexpectedly, topology of Model 4 suggests that unlike RIP1, TRADD-activated caspase-8 neither cleaves RIP1/RIP3 nor is suppressed by RIP3 (Fig. 5B). Thus, the caspase-8 activation can occur independently of RIP3 phosphorylation in RIP1 knockdown cells (Fig. S3B). RIP1 biphasically regulates necroptosis through the basis set of RIP1, RIP3 and pro-caspase-8, while RIP1 limits apoptosis through the basis set of RIP1, TRADD and pro-caspase-8. Finally, we examined whether Model 4 could precisely quantify RIP1 level-determined total cell death, including apoptosis and necroptosis. We experimentally measured the cell death in RIP1 knockdown and RIP1 KO cells. As shown in Fig. 5I, the predicted death rate curves matched well with the experimental data, exhibiting an n-shaped biphasic relationship between RIP1 expression level and cell death.

### Distinct cell death outcomes determined by RIP1 level in TNF signaling complexes

**Figure 6:**
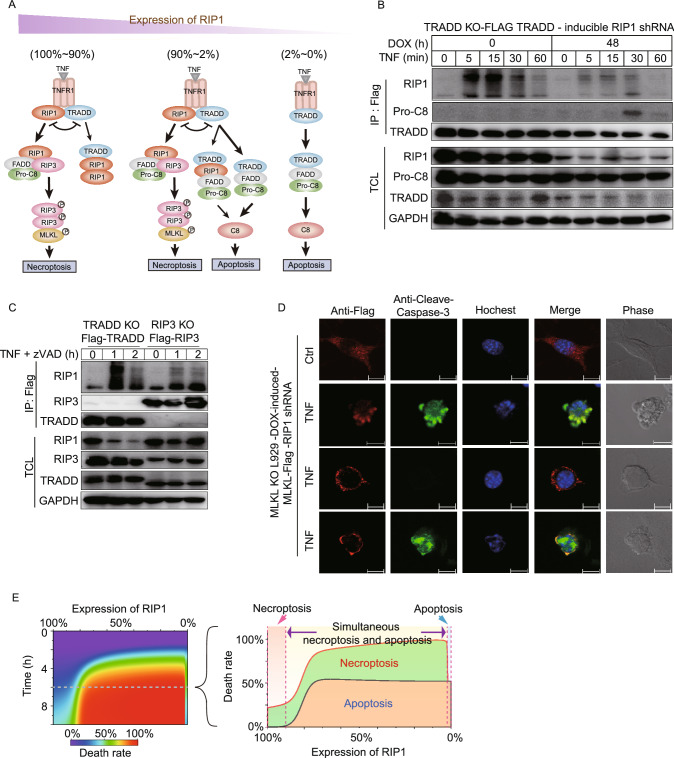
**Distinct cell death outcomes determined by RIP1 level.** (A) Proposed scheme of complexes assembly and cell death types that are quantitatively determined by RIP1 level. The thick and thin arrows denote strong and weak signal transduction processes, respectively. (B) Flag-TRADD-expressing TRADD KO cells were treated with TNF. The cell lysates were immunoprecipitated with anti-Flag antibodies. Immunocomplexes and cell lysates were analyzed by Western blot. (C) Flag-TRADD-expressing TRADD KO cells and Flag-RIP3-expressing RIP3 KO cells were treated with TNF plus zVAD and then were analyzed by Western blot. (D) RIP1 was knocked down in DOX-inducible MLKL-Flag expressing MLKL KO cells. Cells were induced with DOX and were treated with or without TNF. Cells were then fixed and immunostained for Flag and cleaved-caspase-3, counterstained with hochest. Distributions of MLKL in cells are defined as four types: uniformly diffused with or without cleaved-caspase-3, plasma membrane location with or without cleaved-caspase-3. Each scale bar indicates 10 µm. (E) Two-dimensional diagram simulations of RIP1 decrease induced n-shaped cell death rate upon TNF treatment (left panel). Right panel is the contribution proportions of apoptosis (brown area) and necroptosis (green area) after 6 h TNF treatment

Taken together, we can derive a quantitative picture of TNF-induced complexes formation dynamics, signaling transduction and cell fate decisions under different RIP1 expression levels (Fig. 6A). TNF treatment leads to the recruitment of RIP1 and TRADD to TNFR1 in a competitive manner (Fig. S6A and S6B) (Zheng et al., 2006). When RIP1 level is extremely low, i.e., < ~2% of wildtype cells (< ~1000 mpc), TRADD-mediated caspase-8 activation is predominant, which solely leads to apoptosis (Fig. 6A, right panel). Figure 6B shows the recruitment of pro-caspase-8 to TRADD complex in Flag-TRADD-expressing TRADD KO cell line after RIP1 was knocked down. 48 h DOX treatment significantly reduced overall RIP1 level and RIP1 in TRADD complex, which led to pro-caspase-8 recruitment to TRADD (Fig. 6B). It is known that TRADD and RIP1 might be subsequently released either together or separately from TNFR1 (Fig. 6A, left and middle panel) (Zheng et al., 2006). When RIP1 level is within ~2%–90% of wildtype (~1000–46,000 mpc), TRADD- and/or TRADD+-RIP1- dependent apoptosis should occur. Meanwhile, a fraction of RIP1, which is not associated with TRADD, could recruit RIP3 to initiate necroptosis (Fig. 6A, middle panel). This signal branching is supported by the observation that TRADD and RIP3 are located in different complexes, and both complexes contain RIP1 (Fig. 6C). Simultaneous activation of caspase-8 and RIP3 (Fig. S3B) and the suppression of necroptosis by an increase of RIP1 level happen (Fig. 6A, middle panel). Since high RIP1 level eliminates TRADD-induced caspase-8 activation (Fig. 5G), TNF stimulation leads to necroptosis alone when RIP1 level is > ~90% of wildtype (> ~46,000 mpc) (Fig. 6A, left panel).

Whether cell undergoes apoptosis or necroptosis depends on which pathway is dominant. As there is no mutual inhibition between the downstream signaling events of apoptosis and necroptosis in RIP1 knockdown cells (Fig. 6A, middle panel), co-occurrence of apoptosis and necroptosis in an individual cell is possible. Immunofluorescence microscopy data supported such an idea (Fig. 6D).

Hence, RIP1 level is a “switch node” in determining the outcomes of cell death. Consistent with the proposed picture shown in Fig. 6A, simulation result indicated that death rate is low at both extremely low and high RIP1 levels, while high death rate is observed within a broad middle range of RIP1 level (Fig. 6E, left panel). We further plotted the contribution of apoptosis and necroptosis to cell death (Fig. 6E, right panel), showing that the occurrence of apoptosis or necroptosis alone is typical at the range of 0%–2% (0–1000 mpc) or ~90%–100% (~46,000–51,000 mpc) RIP1 levels respectively. Simultaneous death outcome occurs at the range of ~2%–90% RIP1 levels with about equal contribution of apoptosis and necroptosis to total cell death. The equal contribution of apoptosis and necroptosis in TNF-induced cell death is likely to be resulted from the similar increasing tendency of caspase-8 activation (Fig. S4B) and RIP3 phosphorylation (Fig. S5B) to RIP1 level decrease. Counting of apoptotic and necroptotic cells of the experiment shown in Fig. 6D validated the predicted equal contribution in RIP1 knockdown cells (Fig. S6C).

## Discussion

Despite the fact that TNF signaling pathway has been extensively studied, quantitative elucidation of the components, their interactions and their distinct dynamic responses in complexes is still lacking. Here we studied TNF-induced cell death from a quantitative aspect. We employed absolute quantification methods to obtain proteomic data of the dynamic assembling and de-assembling of TNF-induced TNFR1, RIP1, and RIP3 complexes, and then applied these data to mathematical modeling. The SWATH-MS-based model provides an absolutely quantitative picture of TNF-induced cell death signaling, which revealed previously unknown roles of RIP1 level in specifying cell fate decisions.

As a key upstream regulator in determining cell fate, RIP1 level in cells is modulated by various factors. Chen et al. found that geldanamycin can decrease RIP1 level through decreasing the Hsp90 protein level in primary cortical neurons, which might play a role in the prevention of stroke (Chen et al., 2012). RIP1 expression level is also proved to be elevated by baculovirus transduction in mammalian cells (Wang et al., 2017). Besides, a recent study performed by Someda et al. suggested that retinoic acid enhances the expression of RIP1, which stimulates necroptosis (Someda et al., 2020). Deficiency of selenium, one of the essential trace elements in human and animal body, induces the increase of RIP1 expression and promotes tracheal injury through necroptosis (Wang et al., 2020). Therefore, RIP1 level can be modulated by cellular mechanisms, which could play a role in determining cell viability or death and the mode of cell death.

Sufficient RIP3 expression in a given cell originally had no or low RIP3 expression can switch it death from apoptosis to necroptosis (Zhang et al., 2009). Simulated result suggests that the effect of RIP3 level on death rate and mode of cell death is quite different from that of RIP1 level (Fig. S8A). Necroptosis occurs only when RIP3 level is high, and the death rate is first reduced with the decrease of RIP3 and then increases but the death mode converts to apoptosis (Fig. S8A, lower panel). Further analysis (Fig. S8B) indicates that decrease of RIP3 significantly reduces the contribution of necroptosis to cell death when RIP1 level decreases.

The roles of RIP1 in necroptosis are complicated as RIP1 is not only required for necroptosis but also has an inhibitory effect (Weinlich and Green, 2014). Our study demonstrated that the dual roles are RIP1 level-dependent. RIP1 promotes necroptosis within extremely low level range (< ~2% of wildtype) and inhibits necroptosis at higher levels, exhibiting a biphasic regulation of necroptosis by RIP1. Further mechanistic insights unraveled that the biphasic relationship is determined by the nonlinear threshold pattern of RIP3 and linear pro-caspase-8 recruitments to necrosome. The identified threshold pattern indicates that full phosphorylation of RIP3 only requires a very low RIP1 level (~ 2% of wildtype), which is supported by a recent structure study of the RIP1-RIP3 necrosome (Mompean et al., 2018). This pattern is likely because a small amount RIP1-RIP3 hetero-interaction is sufficient to trigger RIP3-RIP3 homo-interaction and the latter can form a higher-order assembly structure of active necrosome (Wu, 2013; Bashor et al., 2019). RIP1 binds to FADD to recruit pro-caspase-8 (Han et al., 2011; Berghe et al., 2014). Here we showed that RIP1 level is linearly associated with the recruitment of pro-caspase-8 to necrosome, which inhibits the signaling toward RIP3 phosphorylation. The result is well in line with previous literature (Oberst et al., 2011; Orozco et al., 2014), suggesting that the proposed mechanism of the triangular relationship among RIP1, RIP3 and caspase-8 in the determination of cell death outcomes might be generally applicable.

The roles of RIP1 in regulating cellular events are known to be regulated by RIP1 protein modifications such as ubiquitylation and phosphorylation (Ofengeim and Yuan, 2013). These modifications affect either RIP1 level or interaction with other proteins or RIP1 kinase activity in different complexes (Dondelinger et al., 2017; Jaco et al., 2017; Xu et al., 2018; Newton et al., 2019a; Newton et al., 2019b). We observed that phosphorylation of RIP1 (Meng et al., 2018) was barely affected when the expression level of RIP1 decreased in cells (Fig. S8C), and the ubiquitination level of RIP1 was significantly reduced with the decrease of RIP1 level in cells (Fig. S6A) but it barely affected NF-κB activation (Fig. S7A). Due to technical limitation, we are unable to dissect the contribution of scaffold and kinase activity of RIP1 in our current study. It is known that the promotion of necroptosis by RIP1 is its kinase activity dependent whereas the suppression is mediated by the scaffold function of RIP1 (Berger et al., 2014; Kaiser et al., 2014). As both functions of RIP1 are closely associated with its abundance in cells, our analyses presented quantitative relations between necroptosis and the combined effect of RIP1 kinase activity and its scaffold function.

A “mutual inhibition” relationship between apoptosis and necroptosis pathways is generally assumed as caspase-8 and RIP3 can completely block the activation of each other (Han et al., 2011; Berghe et al., 2014). The role of caspase-8 in cleaving RIP1/RIP3 is well known. However, our refined model suggested that TRADD-dependent caspase-8 activation does not cleave RIP1/RIP3. Further experiments ascertained the reason that TRADD and RIP3 are located in two distinct complexes, which provides a regulatory mechanism of the co-occurrence of apoptosis and necroptosis in RIP1 knockdown cells. Simultaneous apoptosis and necroptosis was even observed in individual cells. Thus, the relationship between apoptosis and necroptosis pathways is more like “co-existing”, rather than “mutual inhibition”. This finding raises a question how cells make fate decisions in “co-existing” state. We propose that there should be a “speed competition” between apoptosis and necroptosis pathways and the cell fate is determined by the pathway which reaches the final destination first.

Although the biphasic relationship between RIP1 level and cell death is demonstrated in vitro but its relevance to RIP1’s function *in vivo* is unclear. Nonetheless, a few *in vivo* evidences support this finding. The effect of RIP1 knockdown in mouse was evaluated by Suda et al. Decrease of RIP1 markedly exacerbates liver injury through massive apoptosis but not necroptosis (Suda et al., 2016), which could be resulted from TRADD dependent apoptosis presented in our Model 4 (Fig. 5G). Kaiser et al. showed that RIP1-deficient animals are more sensitive to necroptosis and apoptosis in response to diverse stimuli (Kaiser et al., 2014), which could resemble the situation that decrease of RIP1 level increased caspase-8 activation and RIP3 phosphorylation in our Model 4 (Fig. 6E). We believe that due to cell type differences and tissue organ specificities the in vivo role of RIP1 should be very complicated, and the downregulation of RIP1 level added one more layer to the complicity. In conclusion, our study presented comprehensive multi-approach analyses for understanding the roles of RIP1 on cell death determination. We suggest that precise quantitative description of signaling transduction with SWATH-MS-based modeling can provide a deeper insight into signaling dynamics that encode biological decision making.

## Materials and methods

### Cell line and cell culture

HEK293T and mouse fibrosarcoma L929 were obtained from ATCC. RIP1 KO, RIP3 KO, MLKL KO L929, TRADD KO and Caspase-8 KO L929 cells were generated by TALEN or CRISPR/Cas9 methods. The knock-out cells were determined by sequencing of targeted loci and immunoblotting of the expression of respective proteins. All cells were maintained in Dulbecco’s modified Eagle’s medium (DMEM), supplemented with 10% fetal bovine serum, 2 mmol/L L-glutamine, 100 IU penicillin, and 100 mg/mL streptomycin at 37 °C in a humidified incubator containing 5% CO_2_. The target sites were designed as follows: RIP3: “CTAACATTCTGCTGGA”; MLKL: “ATCATTGGAATACCGT”; RIP1: “AACCGCGCTGAGTGAGTTGG”; TRADD: “AAGATGGCAGCCGGTCAGAA”; Caspase-8: “GTGTTCAAATACATACGCCT”.

To generate RIP1 N-terminal Flag knock-in L929 cells, homologous recombination strategy was employed. The rAAV (Adeno-associated virus) targeting vector was constructed by insertion of left homologous arm (about 1kb genomic DNA sequences upstream of the start codon of ripk1) and right homologous arm (about 1 kb genomic DNA sequences downstream of the star codon of ripk1) into the rAAV-Neo-Lox P-flag KI vector. Targeting rAAV virues were packaged in 293T cells. L929 cells were infected with the targeting rAAV virus and then selected for neomycin-resistant clones 24 h post infection. Those clones were then screened for homologous recombination by genomic PCR and the positive clones were infected with adenovirus expressing Cre-recombinase to excise the neomycin gene cassette. The final successful flag knock-in clones were confirmed by genomic PCR.

### Purification of heavy amino acid labeled proteins

293T cells were cultured in amino acid deficient DMEM (Thermo) supplemented with “heavy” 13C6, 15N2 L-Lysine and 13C6, 15N4 L-Arginine (Sigma), 10% dialyzed FBS (Thermo), 0.1 mg/mL streptomycin, and 0.2 U/mL penicillin. Cells were grown for six doubling times prior to use in order for the cells to be fully incorporated with the labeled amino acids. Since overexpression of RIP3 in 293T causes extensive cell death, we synthesize the DNA sequence encoding the peptides of RIP3 that are frequently detected by mass spectrometry. RIP3 fragment DNA and the full-length TRADD DNA sequences were cloned into overexpression vectors that harbor 6× His tag. Overexpression plasmids were transfected into 293T. After 48 h, the cells were lysed with 8 mol/L urea and the target proteins were purified using Ni-NTA column and eluted with 250 mmol/L imidazole. The purified proteins were separated on SDS-PAGE for purity verification (Fig. S1I). The absolute amount of purified proteins was determined by AAA-MS (Table S3).

### Immunoprecipitation and digestion

To purify TNFR1 complexes in L929 cell lines, wildtype L929 cells were treated with 100 ng/mL Flag-TNF for 0, 5, 15, 30, 45 and 60 min, respectively. Flag-knockin RIP1 L929 cells were treated with 10 ng/mL TNF for 0, 5, 30, 60, 120, 180, 240, 300, 360 and 420 min for isolation of RIP1 complexes. Flag-RIP3-reconstituted L929 cells were treated with TNF for 0, 120, 180, 330, 420 min for purification of RIP3 complexes.

Ten 15-cm dish cells were collected for each time point experiment, and three biological replicates were carried out. After 3× Flag-TNF or TNF treatment, cells were immediately washed twice with ice-cold PBS and harvested by scraping. The harvested cells were washed with PBS and lysed for 30 min on ice in HBS lysis buffer (12.5 mmol/L HEPES, 150 mmol/L NaCl, 1% Nonidet P-40, pH 7.5) with the protease inhibitor cocktail. Cell lysates were then spun down at 20,000 ×*g* for 30 min. The soluble fraction was collected, and immunoprecipitated overnight with anti-Flag M2 antibody-conjugated agarose at 4 °C. Resins containing protein complexes were washed three times with HBS lysis buffer. Proteins were subsequently eluted twice with 0.2 mg/mL of 3× flag peptides in HBS lysis buffer for 30 min each time, and elution was pooled for a final volume of 300 μL. Proteins in the elution were precipitated with 20% trichloroacetic acid (TCA) and the pellet was washed two times with 1-mL cold acetone and dried in speedvac.

TCA-precipitated proteins were re-suspended in 50 μL 1% SDC (sodium deoxycholate) in 10 mmol/L TCEP (Tris(2-carboxyethyl)phosphine hydrochloride), 40 mmol/L CAA (chloroacetamide), 100 mmol/L Tris-HCl pH 8.5. The appropriate amount of purified heavy amino acid labeled TRADD was spiked into the TNFR1 and RIP1 IP samples, and purified heavy amino acid labeled RIP3 was spiked into RIP3 IP samples. IP samples were subsequently shaken at 37 °C for 30 min. Protein concentration was measured with 660 nm Protein Assay Reagent (Piece, Thermo). 1% SDC was then diluted to 0.5% SDC with water, and trypsin (Sigma) was added at the ratio of 1:50 (trypsin: protein). The digestion was performed at 37 °C overnight. 1% TFA was added to stop the reactions followed by centrifugation at 20,000 ×*g* for 10 min. The supernatants were subsequently transferred to C18 STAGEtips for desalting. Tryptic peptides were eluted with 70% ACN/1% FA and dried in speedvac.

### Absolute quantification of RIP1 protein amount in individual cells

To obtain the absolute amount of RIP1 in individual L929 cell, the RIP1 peptide (HQAIFDNTTSLTDEHLNPIR) containing “heavy” 13C6, 15N2 L-Lysine and 13C6, 15N4 L-Arginine was purchased from the Go Top Peptide Biotech Company (Hangzhou, China). The number of L929 cells was counted and collected followed by three-time PBS wash. Three biological experiments were performed. Cells were lysed with 1% SDC in 10 mmol/L TCEP (Tris(2-carboxyethyl) phosphine hydrochloride), 40 mmol/L CAA (chloroacetamide), 100 mmol/L Tris-HCl pH 8.5, followed by 37 °C for 30 min. The lysate was cleared by centrifugation. Protein concentration was assayed with 660 nm protein Assay Reagent (Piece, Thermo). 100 μg proteins were subjected to trypsin digestion. The digestion was performed at 37 °C overnight. Heavy amino acid labeled peptide was added into the reactions. 1% TFA was added to stop the reactions followed by centrifugation at 20,000 ×*g* for 10 min. The supernatants were subsequently transferred to C18 STAGEtips for desalting. Tryptic peptides were eluted with 70% ACN/1% FA and dried in SpeedyVac. The L929 cell lysate peptides were dissolved in 0.1% FA and about 5 μg peptides (we assume digestion efficiency is 100%) were measured on mass spectrometry in SWATH-MS mode. The LC gradient time is 180 min, and SWATH-MS setting is 100 VW (See below for detail).

### Mass spectrometry

Peptides were dissolved in 0.1% formic acid and analyzed by mass spectrometry in DDA and SWATH mode. MS analysis was performed on a TripleTOF 5600 (Sciex) mass spectrometry coupled to NanoLC Ultra 2D Plus (Eksigent) HPLC system. Peptides were first bound to a 5 mm × 500 μm trap column packed with Zorbax C18 5-μm 200- Å resin using 0.1% (*v*/*v*) formic acid/2% acetonitrile in H_2_O at 10 μL/min for 5 min, and then separated from 2% to 35% buffer B (buffer A: 0.1% (*v*/*v*) formic acid, 5% DMSO in H_2_O, buffer B: 0.1% (*v*/*v*) formic acid, 5% DMSO in acetonitrile) on a 35 cm × 75 μm in-house pulled emitter-integrated column packed with Magic C18 AQ 3-μm 200- Å resin.

For DDA, 250-ms MS1 scan was performed in the range of 350–1,250 m/z, and up to 20 most intense precursors with charge state 2–5 were isolated for fragmentation, and MS/MS spectra were collected in the range of 100–1,800 m/z for 100 ms.

For SWATH-MS, a 250-ms survey scan (TOF-MS) which was collected in 350–1,500 m/z was performed followed by 32 100-ms or 100 33-ms MS2 experiments which were collected in 100–1,800 m/z. The 32 MS2 experiments used an isolation width of 26 m/z (containing 1 m/z for the window overlap) to cover the precursor mass range of 400–1,200 m/z. The 100 variable isolation windows are “399.5–409.9, 408.9–418.9, 417.9–427.4, 426.4–436, 435–443.6, 442.6–450.8, 449.8–458, 457–464.8, 463.8–471.1, 470.1–476.9, 475.9–482.8, 481.8–488.6, 487.6–494, 493–499, 498–504.4, 503.4–509.3, 508.3–514.3, 513.3–519.2, 518.2–524.2, 523.2–529.1, 528.1–534.1, 533.1–539, 538–543.5, 542.5–548.5, 547.5–553, 552–558, 557–562.5, 561.5–567, 566–571.5, 570.5–576, 575–580.5, 579.5–585, 584–589.5, 588.5–594, 593–598, 597–602.5, 601.5–607, 606–611.1, 610.1–615.6, 614.6–620.1, 619.1–624.6, 623.6–628.6, 627.6–633.1, 632.1–637.6, 636.6–642.1, 641.1–646.6, 645.6–651.1, 650.1–655.6, 654.6–660.1, 659.1–665.1, 664.1–669.6, 668.6–674.5, 673.5–679, 678–684, 683–688.5, 687.5–693.4, 692.4–698.4, 697.4–703.3, 702.3–708.7, 707.7–713.7, 712.7–719.1, 718.1–724.5, 723.5–729.9, 728.9–735.3, 734.3–740.7, 739.7–746.5, 745.5–751.9, 750.9–757.8, 756.8–763.6, 762.6–769.5, 768.5–775.3, 774.3–781.2, 780.2–787, 786–793.3, 792.3–800.1, 799.1–806.4, 805.4–813.1, 812.1–820.3, 819.3–827.5, 826.5–835.2, 834.2–843.3, 842.3–851.4, 850.4–859.9, 858.9–868.9, 867.9–878.4, 877.4–888.3, 887.3–899.1, 898.1–910.3, 909.3–922.9, 921.9–936, 935–949.5, 948.5–963.4, 962.4–978.7, 977.7–994.9, 993.9–1,015.6, 1,014.6–1,042.2, 1,041.2–1,070.1, 1,069.1–1,100.7, 1,099.7–1,140.7, 1,139.7–1,196.5”. Ions were fragmented for MS2 experiment in the collision cell using a collision energy according to the equation of a doubly charged peptide, ramped ±15 V from the calculated collision energy.

### Group-DIA analysis

SWATH wiff files were converted to profile mzXML files with MSconvert (Proteowizard, Version 3.0.4472). The mzXML files were split into 1 MS1 mzML and 32 or 100 MS2 mzML files using in-house scripts. These mzML files were input into Group-DIA analysis. 18 runs in TNFR1 dataset, 30 runs in RIP1 dataset and 17 runs in RIP3 dataset were separately analyzed by Group-DIA. Group-DIA composed of four modules, “alignment”, “analysis”, “identification” and “validation”. For internal spectral library building, only “alignment” and “analysis” modules were performed. MS1 profiles in multiple runs were first aligned, and XICs of precursors and product ions were concatenated for multiple runs. Similarity comparison was performed between the concatenated XICs of precursors and product ions. The precursors and product ions pairs were extracted and stored in mgf and mzML formats.

### Spectral library building

To enable in-depth exploration of SWATH data, we combined internal spectral library and DDA spectral library. Group-DIA-generated mgf files were converted to mzXML files using TPP msconvert (Trans Proteomics Pipeline, Version 4.8). DDA wiff files were converted to centroided mzXML files with qtofpeakpicker (Trans Proteomics Pipeline, Version 4.8). These mzXML files were searched with Comet (Version 2017.01) and X!tandem (Version 2013.06.15.1, native and k-score) against the full non-redundant, canonical mouse genome as annotated by UniprotKB/Swiss-Prot (downloaded in September, 2014) appendant with common contaminants and reversed sequence decoys (33,864 sequences includes decoys). The search parameters were set as followed, parent monoisotopic tolerance 50 ppm, modification 57.021464@C, potential modification 15.994915@M and maximum missed cleavage sites 2. The pep.xml files were validated with PeptideProphet and combined with iProphet. The Mayu (version 1.07) was utilized for FDR estimation. The peptide ions filtered at 1% protein FDR were imported into spectraST for spectral library building. The retention time of peptides in sptxt file was replaced with iRT time using spectrast2spectrast_irt.py script, where ciRT peptides were used for retention time normalization. Subsequently, the sptxt files were made consensus non-abundant sptxt files with spectraST. Sptxt files were converted to Peakview software compatible txt files using spectrast2tsv.py script.

### Targeted analysis of SWATH-MS data using Peakview software

The spectral libraries were converted to Peakview-compatible file. All b-ions from heavy amino acid labeled peptides were filtered out from the library. The parameters of Peakveiw software (Version 2.2) were set as followed. The peptides from the bait proteins and iRT peptides were used for retention time alignment between runs. “Number of transitions per peptide” was “6”, “False Discovery Rate” was “1%”, “XIC Extraction window” was “10 min”, and “XIC width (Da)” was “0.05”.

### Reagents and antibodies

Mouse TNF-α was obtained from eBioscience (San Diego, CA, USA). zVAD was obtained from Calbiochem. Smac mimetic (SM-164) was purchased from APExBIO. Doxycycline hyclate (DOX) and propidium iodide (PI) were obtained from Sigma. Anti-RIP3 (dilution 1:1000), anti-MLKL (dilution 1:1000) and anti-FADD (dilution 1:1000) antibodies were raised using E. coli-expressed GST-RIP3 (287–387 amino acid), GST-MLKL (100–200 amino acids) and GST-FADD (full length), respectively. Anti-IkBαantibody (9242, dilution 1:1000), anti-p38 antibody (9228, dilution 1:1000), anti-p-p38 antibody (9216, dilution 1:1000), anti-JNK antibody (9252, dilution 1:1000), anti-p-JNK antibody (9251, dilution 1:1000), anti-caspase-8 antibody (4790, dilution 1:1000), anti-cleaved caspase-8 antibody (8592, dilution 1:500), anti-caspase-3 antibody (9662, dilution 1:1000) and anti-cleaved caspase-3 antibody (9661, dilution 1:200) were purchased from Cell Signaling Technology. Anti-TRADD antibody (ab110644, dilution 1:500), anti-p-RIP3 antibody (ab222320, dilution 1:500) and anti-p-MLKL antibody (ab196436, dilution 1:1000) were purchased from Abcam. Anti-Gapdh antibody (60004-1-Ig, dilution 1:2,000) was from Proteintech. Anti-RIP1 antibody (610459, dilution 1:1000) was from BD Biosciences. Goat anti-Mouse IgG (H + L) Cross-Adsorbed Secondary Antibody, Alexa Fluor 568 (A-11004, dilution 1:100), Goat anti-Rabbit IgG (H + L) Cross-Adsorbed Secondary Antibody and Alexa Fluor 488 (A-11034, dilution 1:100) were purchased from Invitrogen.

### Lentivirus preparation and infection

For lentivirus production, HEK293T cells were transfected with lentiviral vectors and virus-packing plasmids by calcium phosphate transfection. 8–16 h later cell culture medium was changed. The virus-containing medium was collected 36 h later and added to cells with 10 μg/mL of polybrene, and then centrifuged at 2,500 rpm for 30 min. Infectious medium was changed with fresh medium 12 h later.

### RNA interference

All lentiviral-shRNAs were constructed into pLV-H1-EF1α-puro vector or pLV-H1TetO-GFP-Bsd following the manufacturer’s instruction (Biosettia). The indicated shRNA target sequences were: RIP1 shRNA: 5′-GCATTGTCCTTTGGGCAAT-3′; RIP1 shRNA*: 5′-CCACTAGTCTGACTGATGA-3′.

### Cell death assay

Cell survival rates were determined by flow cytometry with two parameters: plasma membrane integrity and cell size. The plasma membrane integrity was tested by the ability of cells to exclude PI. Cells were trypsinized, collected by centrifugation, washed once with PBS, and resuspended in PBS containing 5 µg/mL PI. The levels of PI incorporation were quantified by flow cytometer (EPICS XL; Beckman Coulter, Fullerton, CA, USA). Cell size was evaluated by forward-angle light scattering. PI-negative cells with a normal size were considered living.

### Immunoprecipitation and Western blot

Cells were seeded in a 100 mm dish and grew to reach confluency. After stimulation, cells were washed by PBS for three times and then lysed with lysis buffer (20 mmol/L Tris-HCl, pH 7.5, 150 mmol/L NaCl, 1 mmol/L Na_2_EDTA, 1 mmol/L EGTA, 1% Triton X-100, 2.5 mmol/L sodium pyrophosphate, 1 mmol/L β-glycerophosphate, 1 mmol/L Na_3_VO_4_) on ice for 30 min. Cell lysates were then centrifuged at 20,000 ×*g* for 30 min. The supernatant was immunoprecipitated with anti-Flag M2 beads at 4 °C overnight. After immunoprecipitation, the beads were washed three times in lysis buffer and the immunoprecipitated proteins were subsequently eluted by SDS sample buffer with 0.15 μg/μL 3× Flag peptide. Linear exposures of Western blot were quantified by laser densitometry and Image J software (Table S7).

### Confocal microscopy

Cells were seeded in 20 mm circular cover glass (NEST). After stimulation, cells were washed by PBS for three times, and then fixed with 3% paraformaldehyde for 10 min at room temperature (RT). Being washed by PBS for 2 times, cells were treated with 0.25% Triton X-100 for 10 min at RT to break the cell membrane. These fixed samples were then washed with PBS and blocked with 3% BSA for 30 min at RT. Removing the blocking buffer, primary anti-Flag (mouse, 1:200) and anti-cleaved-caspase-3 (Rabbit, 1:200) antibodies diluted in blocking buffer were used at 4 °C overnight. Samples were then washed by PBS for three times, stained with goat anti-mouse Alexa Fluor 568 and goat anti-rabbit Alexa Fluor 488 at RT for 1 h. Cells were counterstained with Hochest to visualize nuclei. All images were captured and processed using identical settings in the Zeiss LSM 780 laser scanning confocal microscope with a 63×/1.40 NA oil objective. Duplicate cultures were examined, and similar results were obtained in at least three independent experiments.

### Mathematical models

The biochemical reactions and connectivity of signaling components were described using ordinary differential equations (ODEs). Detailed models descriptions, equations and parameters are included in the Supplementary Materials and Tables S4–6. Models were developed and simulated with MATLAB and Python 3.6.

## Abbreviations

A20, TNF alpha-induced protein 3; cIAP1/2, cellular inhibitor of apoptosis 1/2; DDA, data-dependent acquisition; DIA, data-independent acquisition; DOX, doxorubicin; FADD, Fas-associated death domain protein; Hsp90, heat shock protein 90; IFN, interferon; IP, immunoprecipitation; MAPKs, mitogen-activated protein kinases; MLKL, mixed lineage kinase domain-like protein; NF-κB, nuclear factor κB; ODEs, ordinary differential equations; Ppm1b, protein phosphatase 1B; RIP1, receptor-interacting protein kinase 1; RIP3, receptor-interacting protein kinase 3; shRNA, short hairpin RNA; SWATH-MS, sequential window acquisition of all theoretical fragment ion spectra mass spectrometry; TNF, tumor necrosis factor; TNFR1, TNF receptor 1; TRADD, TNFR1-associated death domain protein; TRAF2, TNF receptor-associated factor 2; TRIF, TIR-domain-containing adapter-inducing interferon-β; XICs, extracted ion chromatograms; ZBP1, Z-DNA-binding protein 1; zVAD, z-Val-Ala-DL-Asp-fluoromethylketone.

## Electronic supplementary material

Below is the link to the electronic supplementary material.

Supplementary material 1 (PDF 2763 kb)

## DATA AND MATERIALS AVAILABILITY

Tables S1–3 and S7, all SWATH-MS raw data and spectral libraries can be accessed at ftp://PASS01429:KS769m@ftp.peptideatlas.org/. All cell lines are available from the corresponding author (J.H.). Zipped mathematical code files of the models to generate the results in this study are available upon request from the corresponding author (J.S.).
