# Supplementary material for: RIP1-dependent linear and nonlinear recruitments of caspase-8 and RIP3 respectively to necrosome specify distinct cell death outcomes
Source: Protein Cell. 2021 Jan 2;12(11):858–76. doi: 10.1007/s13238-020-00810-x (PMC8563874; doi:10.1007/s13238-020-00810-x)
Supplement: Supplementary file 1 — Supplementary material 1 (PDF 2763 kb) [file 13238_2020_810_MOESM1_ESM.pdf]

Supplementary materials

Supplementary Text

Modeling principles

All biochemical reactions in TNF-induced cell death signal transduction (Fig. 2B) are represented by molecule-molecule interactions and enzymatic reactions:

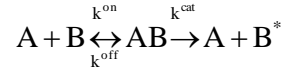

The interactions are mainly described by a reversible reaction step for the association and dissociation of complex partners (AB) with kinetic rate constants k^{on} and k^{off} , respectively. The subsequent protein modification is described by the irreversible catalytic step with the rate constant k^{cat} . Biochemical reaction rates are dependent on protein concentrations and kinetic rate constants according to the law of mass action. Dynamics of the biochemical reactions and connectivity of signaling molecules can be formulated as a set of coupled ordinary differential equations (ODEs):

$$dx_i / dt = \sum_{j=1}^n v_{ij} \cdot q_j, (i = 1, \dots, m)$$

where m represents the number of components with the concentration x_i and dx_i/dt is the rate of concentration change with time. n is the number of reactions with the rate q_j , and v_{ij} denotes the element of stoichiometric matrix that links the reaction rates of q_i with component x_i . A detailed description of the models and ODEs are given in Table S5. Besides the association and dissociation constants, the kinetic parameters also include Michaelis-Menten constants, turnover numbers and so on. The kinetic parameters (Table S6) are reasonably estimated with biochemical constraints and were mostly fixed with a global optimization method that minimizes the deviation between simulation results and SWATH-MS data of the key proteins behavior shown in Fig. 2C and Fig. S2. Initial concentrations of the compounds are mostly measured by using SWATH-MS or partially obtained from earlier literature (Table S5).

Detail mechanistic model description

To systematically explore the emergent intricate properties of RIP1 in cell death, we developed the mathematical models that incorporated protein interactions, phosphorylation and enzymatic reactions. The models are formulated by a set of coupled ODEs, describing the time evolution of concentrations of components in complexes. The integrative models of the RIP1 network are composed of three modules: the complex I formation module, the necrosome activation module, and the mutual inhibition interaction between apoptosis and necroptosis pathways. The details of each module are presented as follows.

Complex I formation module

Upon TNF binding to its receptor (TNFR1), the key functional proteins, TRADD and RIP1 are subsequently recruited to form complex I, which is a pedestal for activation of the downstream effector proteins (Brenner et al., 2015). Two signaling events take place between TNF and TNFR1. The first event occurs when TNF binds to TNFR1 at the plasma-membrane and forms the TNF-TNFR1 complex, which can recruit TRADD and RIP1 through the TNFR1 Death-Domain (Zheng et al., 2006). The second event occurs when TNFR1 is subsequently modified and dropped from the TNF-TNFR1 complex. These two events can be described by the following biochemical reactions:

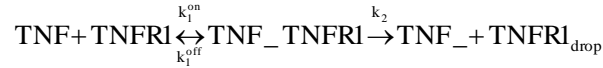

TNF-TNFR1 complex further recruits the TRADD and RIP1 to form complex I, and the process can be described by the following steps. Both TRADD and RIP1 are bound to TNF-TNFR1, forming the TNF-TNFR1-TRADD and TNF-TNFR1-RIP1 complexes, respectively. SWATH-MS data indicated that the ratio of the three proteins (TNFR1, TRADD, and RIP1) in complex I is approximately 1:1:1 after 5 min TNF treatment (Fig. 2C), suggesting that there are at least two binding sites for each TNFR1. Hence, the constitutions of complex I could be TNF-TNFR1-TRADD-TRADD, TNF-TNFR1-RIP1-RIP1, TNF-TNFR1-TRADD-RIP1, and TNF-TNFR1-RIP1-TRADD. We only considered the TNF-TNFR1-TRADD-TRADD and TNF-TNFR1-RIP1-RIP1 constitutions to obtain a model of lower complexity, which is sufficient to reproduce the observed dynamics. Experimental observations indicate that TRADD and RIP1 compete for recruitment to TNFR1 (Zheng et al., 2006), which can also be reproduced by the considered constitutions (Figs. S6A and B). TRADD and RIP1 could subsequently drop from complex I, which is an essential step for the activation of the downstream signaling components. Thus, the multiple signaling steps can be depicted by the following reactions:

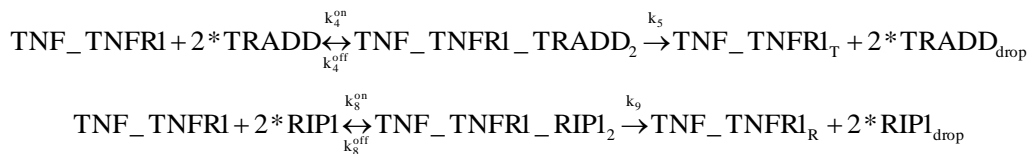

We also made an assumption that the dropped components ($\text{TNFR1}_{\text{drop}}$, $\text{TRADD}_{\text{drop}}$ and $\text{RIP1}_{\text{drop}}$) could turn into the initial states in some cases. The reactions are described by the following reactions:

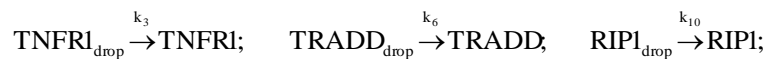

Taken together, based on the reactions above, the dynamics of complex I formation could be formulated as a set of coupled ODEs. The following are representative derivations of TNFR1 and TNF_TNFR1 complex of the all ODEs:

$$\begin{aligned}\frac{dT_{NFRI}}{dt} &= -k_1^{on} \cdot TNF \cdot TNFRI + k_1^{off} \cdot TNF\_TNFRI + k_3 \cdot TNFRI_{drop}; \\ \frac{dT_{NF\_TNFRI}}{dt} &= k_1^{on} \cdot TNF \cdot TNFRI - k_1^{off} \cdot TNF\_TNFRI - k_2 \cdot TNF\_TNFRI - k_4^{on} \cdot TNF\_TNFRI \cdot TRADD^2 \\ &\quad + k_4^{off} \cdot TNF\_TNFRI\_TRADD_2 - k_8^{on} \cdot TNF\_TNFRI \cdot RIP1^2 + k_8^{off} \cdot TNF\_TNFRI\_RIP1_2;\end{aligned}$$

All the reactions and ODEs of the complex I forming dynamics included in the models are presented by Reactions V1~V11 in Table S4 and Equations E1~E12 in Table S5. The related parameters are presented in Table S6.

Necrosome activation module

Disassociation of TRADD and RIP1 from complex I subsequently forms the cytosolic death-initiating signaling complexes (Brenner et al., 2015), called necrosome. Necrosome contains both necroptotic and apoptotic signaling, depending on whether RIP3 is expressed in cells (Zhang et al., 2009; Han et al., 2011; Brenner et al., 2015). The necroptotic signaling is activated through the association of RIP1 and RIP3 via their RIP homotypic interaction motif (RHIM) domains (Zhang et al., 2009; Han et al., 2011). The dynamics of disassociated RIP1 from complex I binding to RIP3 is characterized by Reaction V16 in Table S4. RIP1 and RIP3 become phosphorylated on multiple sites either by auto-phosphorylation or cross-phosphorylation, which is required for activating the downstream events. Phosphorylation of RIP3 after binding to RIP1 is a complex process, which is described in an amount dependent pattern of RIP3 binding to RIP1 by Reaction V17. Necroptosis is restricted by Ppm1b which negatively regulates RIP3 phosphorylation in necrosome (Chen et al., 2015). We assumed that Ppm1b restrains RIP3 to bind to RIP1 in the model (Reaction V23). Phosphorylated RIP3 mediates the downstream events of MLKL phosphorylation and ultimately initiates necroptosis (Cai et al., 2014). The processes of phosphorylated RIP3 recruiting MLKL and MLKL phosphorylation are represented by Reactions V24~V26.

The main components for apoptotic signaling in necrosome are discrepant in different scenarios. Previous findings suggested that the apoptosis can be initiated by two distinct caspase-8 activation pathways, i.e., the TRADD- and RIP1-dependent pathways (Wang et al., 2008). RIP1-depletion in cells switches cell death from necroptosis to apoptosis, while a combined knockdown of RIP1 and TRADD completely blocks TNF-driven apoptosis (Vanlangenakker et al., 2011). This observation indicates that apoptotic signaling is TRADD-dependent and acts through the TRADD-FADD-pro-caspase-8 activation processes. Thus, we assumed that in Model 1 the apoptotic components mainly contain TRADD, FADD, and pro-caspase-8. TRADD-dependent apoptotic activation is mainly described by Reactions V28~V30, which represent the processes of TRADD binding to FADD and FADD mediating caspase-8 activation. Nevertheless, either using a mutant form of RIP1 or depleting RIP3 in cells switches the TNF-induced necroptosis to a

RIP1-dependent apoptosis (Duprez et al., 2012; Remijnsen et al., 2014), suggesting that the apoptotic scaffold components primarily contain RIP1, FADD and pro-caspase-8. We therefore considered that the apoptotic signaling is RIP1-dependent in Model 2. The dynamic processes of RIP1-mediated apoptotic activation are characterized by Reactions V12~V15. Indeed, induction of apoptosis in RIP3-depleted cells could be inhibited but not completely abolished by Nec-1 under TNF plus Smac mimetic treatment (Remijnsen et al., 2014), suggesting that TRADD and RIP1 could simultaneously participate into the initiation of apoptosis. The observations indicate that a mixed platform formed by TRADD-RIP1-FADD-pro-Caspase-8 can also be assumed as the main signaling for apoptosis. The TRADD-RIP1-dependent activation processes of apoptosis were constructed in Model 3, which are depicted by Reactions V12~V15 and V28~V30.

Activated caspase-8 subsequently binds to the effector caspases, such as caspase-3 (Reactions V27 and V31), and progresses substrate cleavage, leading to apoptosis. Overall, since there are three possible apoptotic signaling, we separately included them into the whole TNF signaling pathway model, namely Model 1, 2 and 3. Reactions V28~V30 are only included in Model 1 and Model 3, while Reactions V12~V15 are involved in Model 2 and Model 3.

Mutual inhibition between apoptosis and necroptosis

The relationship between apoptotic and necroptotic signaling seems to be linked by caspase-8 and RIP3. It is reported that the caspase-8 prevents RIP3-dependent necroptosis without inducing apoptosis (Tummers et al., 2017). The catalytic activity of caspase-8 can cleave phosphorylated RIP3 and the detailed mechanism remains elusive. Besides, RIP3-deficient cells induce caspase-8 activation and apoptosis occurrence under TNF treatment (Remijnsen et al., 2014), indicating a suppressive role of RIP3 on apoptosis through inactivating caspase-8. The mutual inhibition between RIP3 and caspase-8 indicates a competitive relationship between necroptosis and apoptosis, which is characterized by Reactions V18, V19, V32 and V33 in our models. Complete modeling reactions and ODEs are presented in Tables S4 and S5.

Parameters values and initial amounts selection

There are 57, 59 and 77 total parameters in Model 1, 2 and 3, respectively. All the parameters are firstly restricted to be within the typical biological ranges according to the reaction type. Then we further estimated the parameters based on the experimental data or earlier literature (Albeck et al., 2008). The parameters are mostly determined by a global optimization method that minimizes the deviation between simulation results and SWATH-MS data. The deviation is characterized by using the correlation coefficient, R-square, which is determined as the following functions:

$$R^2 = 1 - \frac{\sum_{i=1}^n (y_{\text{exp}}(t_i) - y_{\text{sim}}(t_i))^2}{\sum_{i=1}^n (y_{\text{exp}}(t_i) - \overline{y_{\text{exp}}})^2}$$

where $y_{\text{exp}}(t_i)$ and $y_{\text{sim}}(t_i)$ are the MS data and simulated data at time t_i , respectively. $\overline{y_{\text{exp}}}$ is the average value of the MS data. As shown in Fig. 2C, the MS data of 8 key proteins are selected to optimize the parameters of the three models. For each protein, there are 5-10 time points. After optimization of parameters, we calculated the average R^2 of the 8 proteins. As a result, the averaged R^2 of each model is 0.95, 0.95 and 0.92 respectively (Fig. 2D). The parameters that are not available from MS data are derived from literature or estimated within a biologically plausible range (Albeck et al., 2008). All the parameters descriptions and values are presented in Table S6. Initial amounts of the proteins in one cell are also required in our models. For most proteins, initial amounts are measured using MS, while the others are obtained from earlier literature or estimation (Albeck et al., 2008). Initial amounts of all the components are listed in Table S5 and the unit is molecules per cell (mpc).

Simplified models for random parameter analysis

Studies have shown that realistic cellular control networks can be decomposed into simple regulatory motifs that carry out specific functions (Ma et al., 2009). Different topologies of the motifs might result in executing different biological functions. In our study, the only difference among the three models is the apoptotic signaling activation process. To uncover the structural and functional discrimination of three models, we simplified the three full models (Fig. 2B) into three simplified models (Fig. S7B), which represent the basic information-processing modules with four key proteins, TRADD, RIP1, caspase-8, and p-RIP3. The simplified models remain the basic functions of the full models: the mutual inhibition between TRADD and RIP1 (the competitive relation between the two proteins to bind TNFR1 in models); RIP1-dependent phosphorylation of RIP3; the mutual inhibition between phosphorylated RIP3 and caspase-8; and the caspase-8 activation dependence on different upstream proteins. Caspase-8 activation, in turns, depends on TRADD, RIP1 and TRADD-RIP1 in simplified models. The inhibition effect of caspase-8 on RIP1 can be considered into the mutual inhibition between pRIP3 and caspase-8 for simplified models analysis (Fig. S7B). The reason is that caspase-8 only cleaves the RIP1 that activates RIP3, rather than the RIP1 that activates caspase-8 (Fig. 2B).

The full ODEs corresponding to the three simplified models are listed as below, in which the reaction rates take the form of Hill function equations:

$$\frac{d[\text{TRADD}]}{dt} = -k_1 \cdot \frac{[\text{RIP1}]}{[\text{RIP1}] + K_1} \cdot [\text{TRADD}] \quad (1)$$

$$\frac{d[\text{RIP1}]}{dt} = -k_2 \cdot \frac{[\text{TRADD}]}{[\text{TRADD}] + K_2} \cdot [\text{RIP1}] - k_3 \cdot \frac{[\text{ProC8}]}{[\text{ProC8}] + K_3} \cdot [\text{RIP1}] \quad (2)$$

$$\frac{d[\text{RIP3}]}{dt} = k_{\text{RIP1}} \cdot \frac{[\text{RIP1}]}{[\text{RIP1}] + K_{\text{RIP1}}} - k_4 \cdot \frac{[\text{ProC8}]}{[\text{ProC8}] + K_4} \cdot [\text{RIP3}] \quad (3)$$

$$\frac{d[\text{ProC8}]}{dt} = k_{\text{TRADD}} \cdot \frac{[\text{TRADD}]}{[\text{TRADD}] + K_{\text{TRADD}}} - k_5 \cdot \frac{[\text{RIP3}]}{[\text{RIP3}] + K_5} \cdot [\text{ProC8}] \quad (4a)$$

$$\frac{d[\text{ProC8}]}{dt} = k_{\text{RIP1}} \cdot \frac{[\text{RIP1}]}{[\text{RIP1}] + K_{\text{RIP1}}} - k_5 \cdot \frac{[\text{RIP3}]}{[\text{RIP3}] + K_5} \cdot [\text{ProC8}] \quad (4b)$$

$$\frac{d[\text{ProC8}]}{dt} = k_{\text{TRADD}} \cdot \frac{[\text{TRADD}]}{[\text{TRADD}] + K_{\text{TRADD}}} + k_{\text{RIP1}} \cdot \frac{[\text{RIP1}]}{[\text{RIP1}] + K_{\text{RIP1}}} - k_5 \cdot \frac{[\text{RIP3}]}{[\text{RIP3}] + K_5} \cdot [\text{ProC8}] \quad (4c)$$

ODEs of (1) - (3) are involved in the three simplified models. While ODEs of (4a), (4b) and (4c) are severally contained in simplified Model 1, Model 2 and Model 3 to describe the distinct caspase-8 activation processes.

To explore the discrimination of the three simplified models with random parameter sets, we restrained the parameters within proper regions. Both of the simplified Model 1 and Model 2 have 14 parameters: 7 reaction rate parameters (k_i) and 7 dissociation constants (K_i). While 16 parameters are involved in simplified Model 3. The majority of reaction rate parameters (k_i) and dissociation constants (K_i) were set up within broad biologically ranges of $10^{-5} \sim 10^3$ mpc/s and $10^2 \sim 10^5$ mpc (Ma et al., 2009). Experimental results indicate that TRADD induces a rapid caspase-8 activation process while RIP1 mediates a very slow process (Vanlangenakker et al., 2011; Remijnsen et al., 2014), suggesting that the reaction parameters of k_{TRADD} should be much larger than k_{RIP1} . Thus, we constrained the TRADD mediated (k_{TRADD}) and RIP1 mediated (k_{RIP1}) caspase-8 activation reaction parameters within the ranges of $10 \sim 10^3$ mpc/s and $10^{-5} \sim 10^{-3}$ mpc/s, respectively. Initial amount of TRADD, RIP1, activated caspase-8, and phosphorylated RIP3 are set as the same values in the full models (Table S5).

We generated random parameter sets within the biologically realistic strength range through a Monte Carlo approach. For each of the three simplified models, 10^6 parameter sets are sampled within the corresponding restrained ranges by using the Latin hypercube sampling method. To characterize the resulting behavior in terms of a variation of RIP3 phosphorylation when RIP1 level decreases, we calculated the difference of RIP3 phosphorylation ($\Delta p\text{-RIP3}$) between 100% RIP1 (wildtype) and 50% RIP1 expression at 2 hours for each parameter set (Fig. 3B). The difference of RIP3 phosphorylation has three possible outcomes: positive number ($\Delta p\text{-RIP3} > 0$), negative number ($\Delta p\text{-RIP3} < 0$), or almost zero ($\Delta p\text{-RIP3} \approx 0$), which correspond to the mechanisms that RIP1 positively, negatively, or does not regulates RIP3 phosphorylation. The results can be mapped on the two-dimensional of $\Delta p\text{-RIP3}$ versus number of corresponding parameter sets statistical distribution. We separately calculated the three outcomes and the corresponding results are shown in Fig. 3C

and Fig. S7C. For each outcome, all the set numbers of the three models are also counted. Percentages of the set numbers of each outcome in total parameter sets which is referred to as the probability are presented in Fig. S7D, respectively.

Moreover, the Gillespie Stochastic Simulation Algorithm (SSA) was also employed to study the dynamical discrimination of the three simplified models (Gillespie, 1977). For simplification, individual cells were treated as independent systems, and proteins were considered to be evenly distributed in cells. Using Gillespie algorithm to simulate the reactions within cells, determining the reaction path and the reaction time are the key steps.

The reaction path is determined by the equation: $\sum_{v=1}^{\mu} a_v = S(\mu) > r_1 a_0$.

While the reaction time is characterized by: $\tau = \frac{1}{a_0} \ln\left(\frac{1}{r_2}\right)$, where $a_0 = \sum_{v=1}^M a_v = \sum_{v=1}^M h_v c_v$.

h_v is the state function of the reaction. When the system is at the state of $(X_1, X_2, \dots, X_N)$ and the reaction is $X_1 + X_2 \rightarrow \text{production}$, h_v equals to $X_1 X_2$.

In above equations, v and μ represent the number of each reaction. $S(\mu)$ is the sum of probability of the reactions with the number that are smaller than or equal to μ . c_v corresponds to the reaction parameter, and r_1 and r_2 are the random numbers which are in a uniform distribution between 0 and 1.

There are 7, 7, and 8 reactions respectively in SModel 1, SModel 2 and SModel 3. The reactions are presented below:

- i. $\text{RIP1} + \text{TRADD} \xrightarrow{c_1} \text{RIP1}$; (Inhibition of TRADD by RIP1)
- ii. $\text{TRADD} + \text{RIP1} \xrightarrow{c_2} \text{TRADD}$; (Inhibition of RIP1 by TRADD)
- iii. $\text{TRADD} \xrightarrow{c_3} \text{TRADD} + \text{C8}$; (Activation of C8 by TRADD)
- iv. $\text{RIP1} \xrightarrow{c_4} \text{RIP1} + \text{C8}$; (Activation of C8 by RIP1)
- v. $\text{RIP1} \xrightarrow{c_5} \text{RIP1} + \text{RIP3}$; (Activation of RIP3 by RIP1)
- vi. $\text{C8} + \text{RIP1} \xrightarrow{c_6} \text{C8}$; (Inhibition of RIP1 by C8)
- vii. $\text{C8} + \text{RIP3} \xrightarrow{c_7} \text{C8}$; (Inhibition of RIP3 by C8)
- viii. $\text{RIP3} + \text{C8} \xrightarrow{c_8} \text{RIP3}$; (Inhibition of C8 by RIP3)

All above reactions are included in SModel 3. Reaction iv is not involved in SModel 1 and reaction iii is not considered in SModel 2.

The calculation steps are as follows:

- (1) Input the corresponding values of the reaction parameters c_v and the initial amounts of the N proteins X_i at the initial time $t_0=0$.
- (2) Calculate a_v and a_0 . $a_v = c_v h_v$.

(3) Generate the random parameters of r_1 and r_2 and determine the reaction path μ and calculate the reaction time τ .

(4) Update the system state at $t=t+\tau$ and back to step (2) for further calculation.

Based on a published literature (Albeck et al., 2008), the parameter ranges of reactions c_1 , c_2 , c_6 , c_7 and c_8 were set up within $10^{-9} \sim 10^{-3} \text{ mpc}^{-1} \text{ s}^{-1}$. While c_3 , c_4 and c_5 were set within the ranges of $10^{-3} \sim 10^{-2}$, $10^{-4} \sim 10^{-3}$ and $10^{-4} \sim 10^{-2} \text{ s}^{-1}$, respectively. Initial amounts of the proteins were set within the same ranges as the random parameter analysis. 10^4 times of simulations were carried out for each simplified model and the probability of the corresponding outcomes were presented in Fig. S7E. The results are consistent with the random parameter analysis (Fig. 3D), indicating that SModel 2 is more robust to achieve the negative regulation of RIP3 phosphorylation by RIP1.

Quantification of Western blotting results

All the Western blots are quantified and analyzed by using the NIH Image J software. The quantified blots data are normalized to GAPDH expression. RIP3 phosphorylation is normalized to the expression level of the total RIP3. All quantification data of the bolts are respectively shown in Table S7.

Parameter estimation for Model 4

Model 2 is further refined by including an additional TRADD-dependent caspase-8 activation, which can subsequently activate the downstream signaling of caspase-3. The refined model is named as Model 4. The added reactions (i.e., Reactions V28~V30) are presented in Table S4. The parameters in Model 4 are either taken from Model 2 if there is a same parameter in both model, or are estimated based on the quantified Western blotting data of caspase-8 in RIP1 KO cells (Fig. 5D, right panel and Fig. S5A) and published models as shown in Table S6.

Cell death rate, which is a comprehensive phenotype, is mainly determined by the downstream effector proteins, MLKL phosphorylation and caspase-3 activation. Thus, dynamic behavior of cell number (N) can be simply characterized by the following ODE:

$$\frac{dN}{dt} = k_a \cdot N - k_d \cdot N^2 - k_{\text{Caspase-3}} \cdot [\text{Caspase-3}] \cdot N - k_{\text{MLKL}} \cdot [\text{MLKL}_{\text{pho}}] \cdot N$$

where N is the population of cells and dN/dt is the population change rate with time. k_a and k_d represent the reaction rates of inherent cells growth and decay. $k_{\text{Caspase-3}}$ and k_{MLKL} denote the reaction rates that introduce the effect of the two effector proteins on N . Initial N is set as 2.0×10^7 cells per dish based on experiments and the four parameters (k_a , k_d , $k_{\text{Caspase-3}}$ and k_{MLKL}) are determined by fitting the experimental cell death rate

curve of wildtype cells (Fig. 5I). Corresponding parameters values are shown in Table S6.

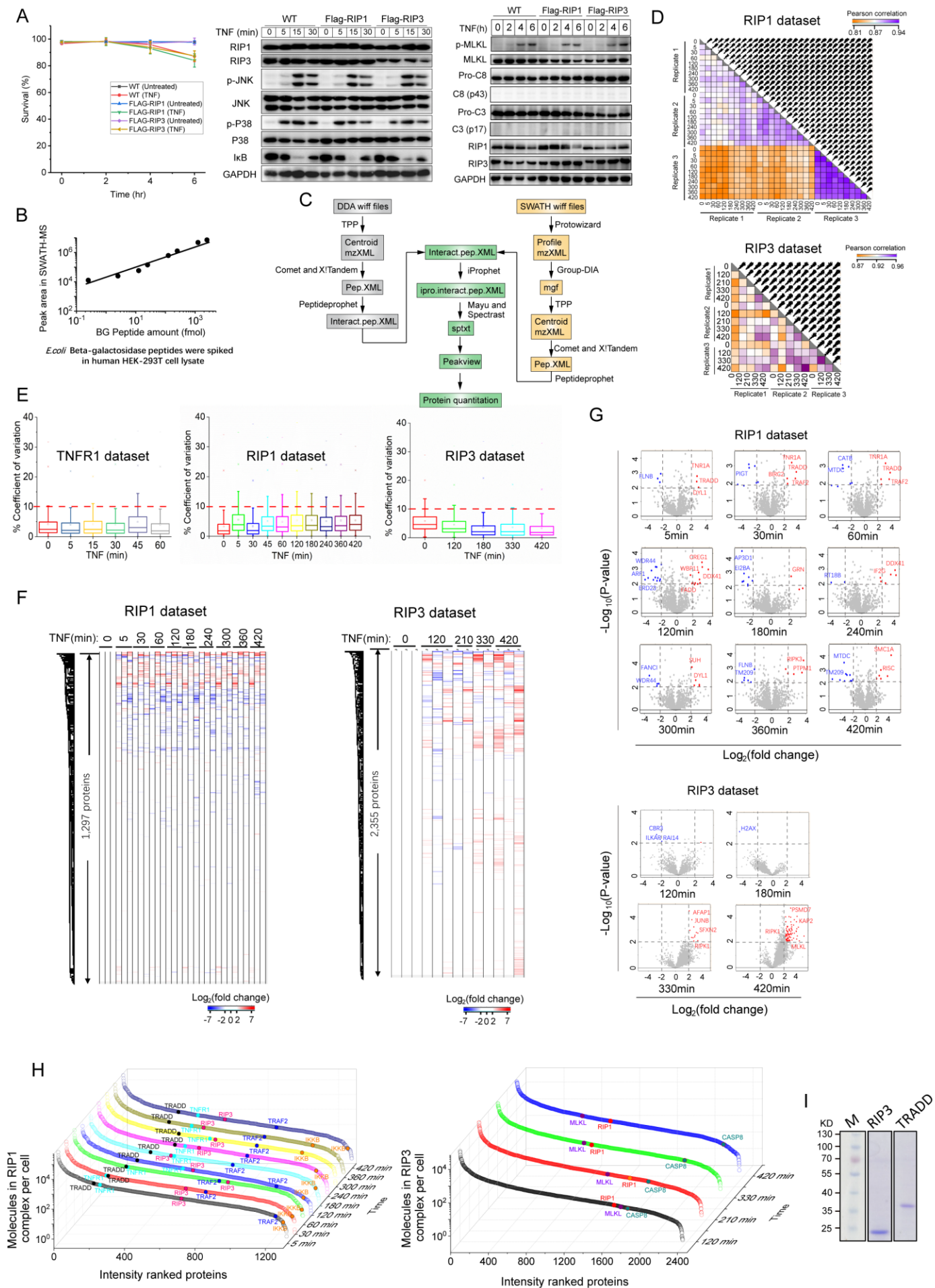

Figure S1. Quantifying TNF signaling complexes using IP-SWATH workflow.

(A) Similar phenotypes of Flag-knockin RIP1 (Flag-RIP1) and Flag-RIP3 reconstituted (Flag-RIP3) cells with wildtype (WT) cells in response to TNF treatment.

(B) Dynamic range of the IP-SWATH workflow. The indicated amount of commercial *Escherichia coli* β -galactosidase peptides were spiked into human HEK-293T cell lysate, followed by trypsin digestion. The tryptic peptides were analyzed using SWATH-MS. Each sample was analyzed in technical triplicates. The β -galactosidase peptide VDEDQPFPAVPK was used for targeted analysis of SWATH-MS data using Peakview software. Each cycle represents the average values of sum of three product ion XIC areas.

(C) Bioinformatic processing procedures of SWATH-MS data. Three software including Trans-Proteomic Pipeline (TPP) (Version 4.8), Group-DIA and Peakview (Version 2.2) were used to analyze SWATH-MS data. 1% protein FDR cutoff was applied for spectral libraries generation. 1% FDR was set for Peakview software analysis.

(D) Reproducibility analysis of quantitative protein intensities in RIP1 dataset (upper panel) and RIP3 dataset (down panel). The protein intensities in two samples were compared in each dataset, and Pearson correlations were calculated.

(E) Quantitative reproducibility of protein intensities in biological triplicates of the TNFR1, RIP1 and RIP3 datasets. The coefficient of variation, which is calculated by dividing the standard deviation of protein profiles by the mean, is reported as percentages.

(F) Heat map of quantitative proteins in RIP1 dataset (left panel) and RIP3 dataset (right panel).

(G) Differential expression analysis of proteins at each timepoint in RIP1 dataset (upper panel) and RIP3 dataset (down panel). Proteins with $|\text{Log}_2(\text{fold change})| > 2$ and $-\text{Log}_{10}(\text{P-value}) > 2$ were considered significantly changed. Up-regulated proteins were labeled in red, and down-regulated proteins were labeled in blue.

(H) Dynamic range of all identified protein amounts per cell in RIP1 dataset (left panel) and RIP3 dataset (right panel). Some interaction proteins were highlighted.

(I) Coomassie brilliant blue R250 staining of purified heavy amino acid labeled TRADD and RIP3.

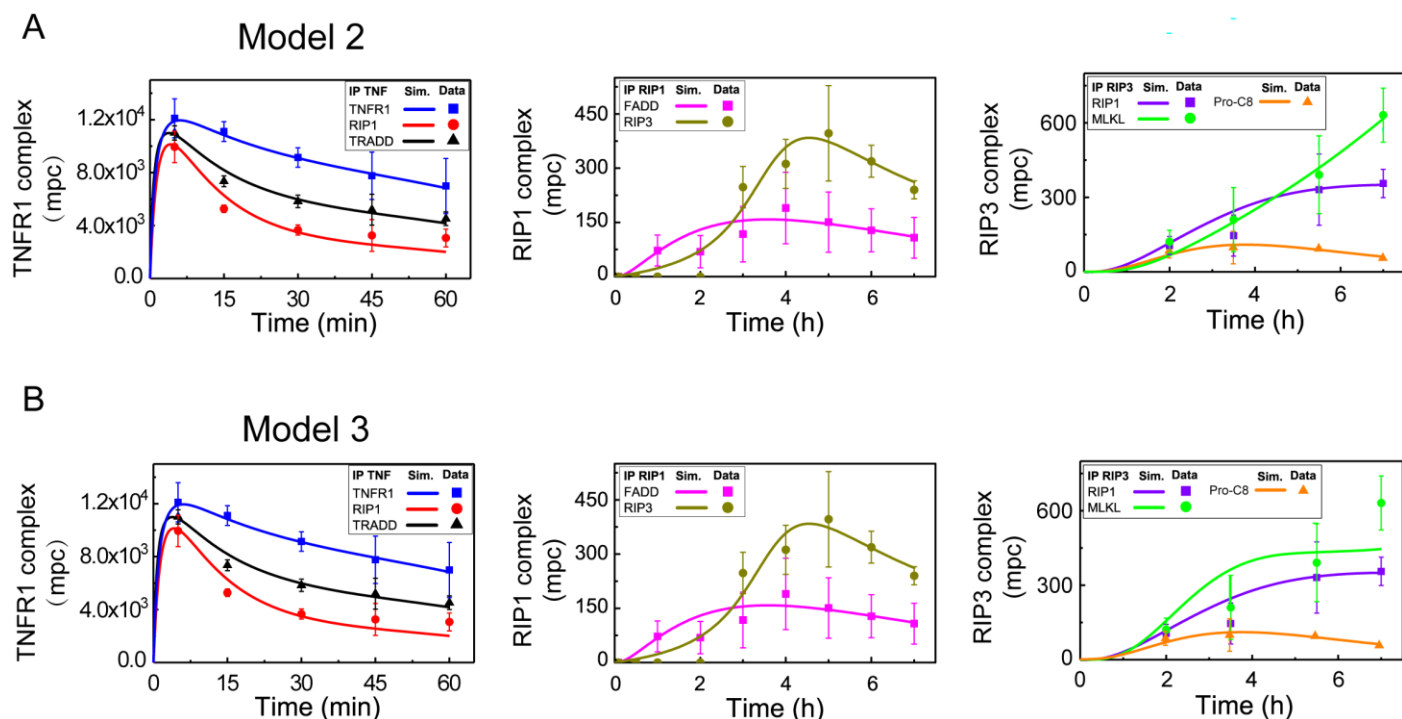

Figure S2. Quantitative modeling of TNF-induced cell death signal transduction.

(A-B) Simulation results with Model 2 (A) and Model 3 (B) to fit SWATH-MS data (dots) of the time-course responses. Error bars denote standard deviation (SD) for three independent experiments.

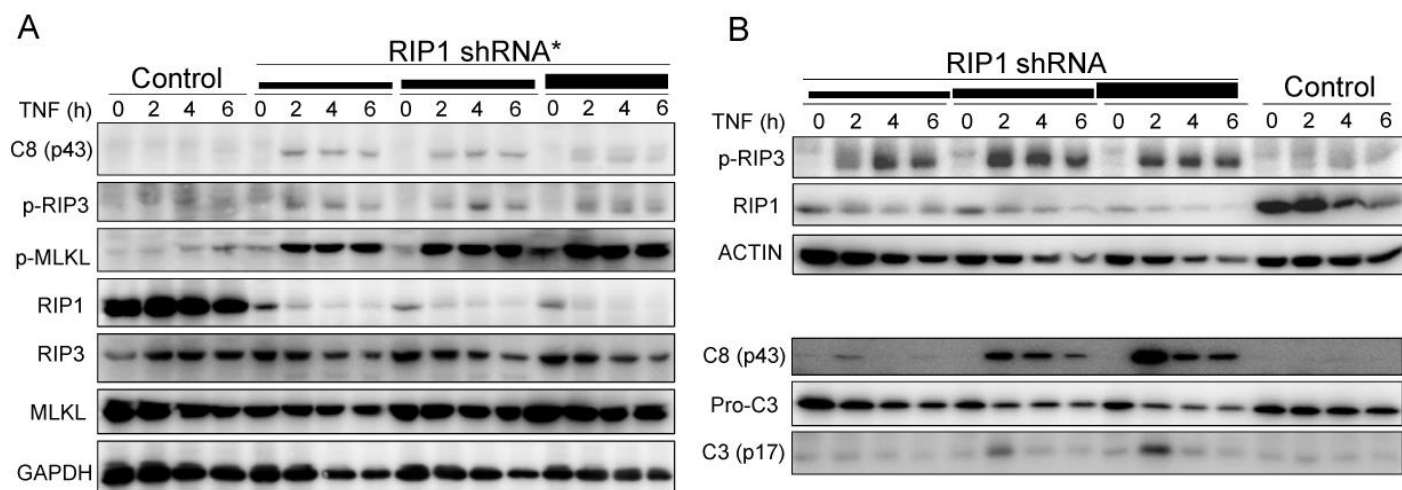

Figure S3. Roles of RIP1 in regulating p-RIP3 and caspase-8 activation.

(A) RIP1 was knocked down to different levels by infecting with different doses of lentivirus-encoding a RIP1 shRNA (RIP1 shRNA*), which is different from the one used in Fig. 3F in cells. 48 hours after infection, the cells were treated with TNF for indicated periods of time and then analyzed by Western blotting with the indicated antibodies.

(B) A replicate experiment of the effect of RIP1 decrease on p-RIP3 and caspase-8 activation.

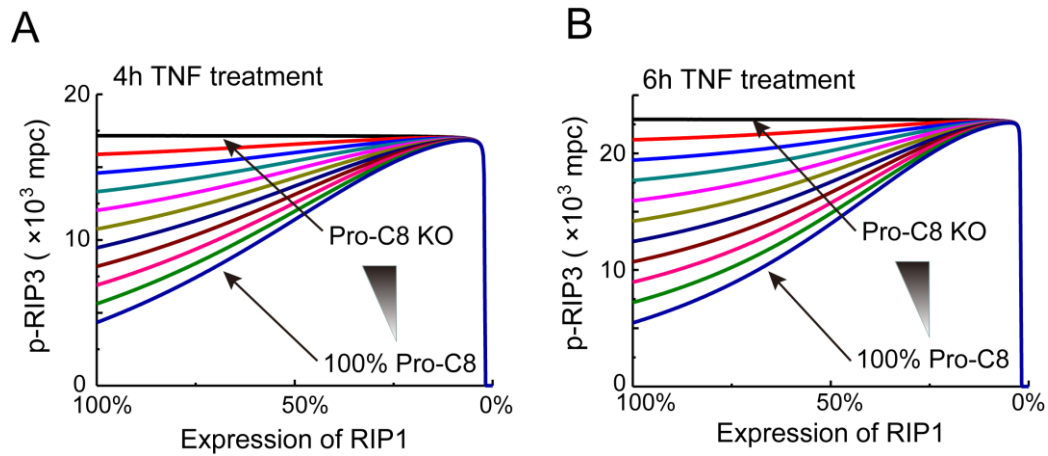

Figure S4. Role of pro-caspase-8 in p-RIP3 negatively regulated by RIP1.

(A-B) Simulated effects of progressive decrease of Pro-C8 expression on RIP1 decrease-induced increase of p-RIP3 at 4 hours (A) and 6 hours (B) of TNF treatment. The arrows below indicate the result with 100% Pro-C8 and the arrows above denote 0% Pro-C8 (Pro-C8 KO).

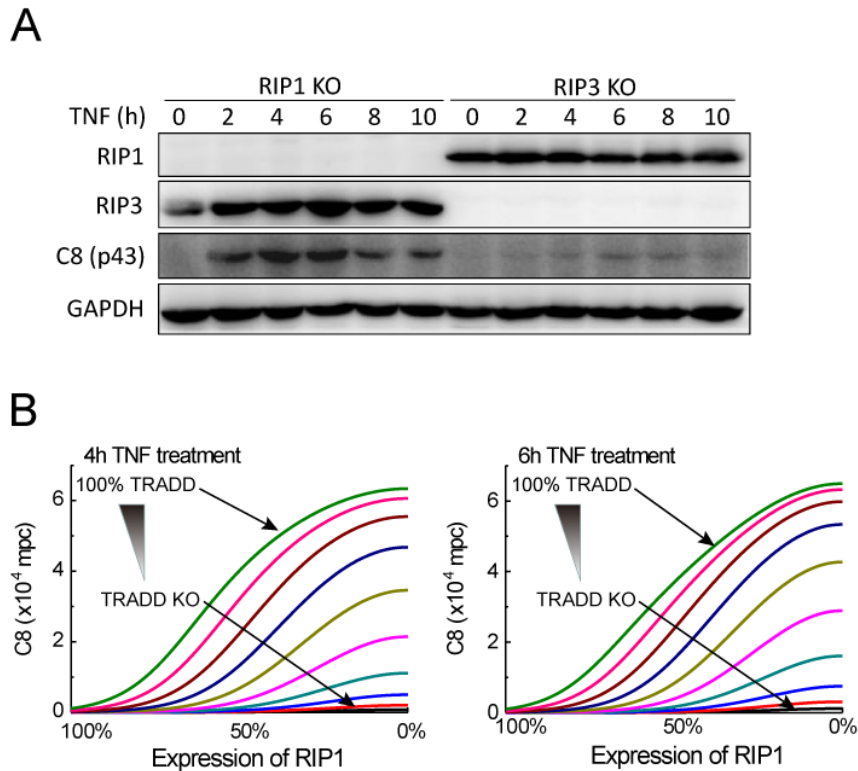

Figure S5. RIP1 suppresses TRADD-dependent caspase-8 activation.

(A) RIP1 KO and RIP3 KO cells were treated with TNF for indicated periods of time and then were analyzed by Western blotting with the indicated antibodies.

(B) Simulated effects of TRADD level reduction on RIP1 decrease induced increase of caspase-8 activation (as indicated by C8 (p43) generation) at 4 hours (left panel) and 6 hours (right panel) of TNF treatment. The arrows above indicate the result with 100% TRADD (WT) and the arrows below denote 0% TRADD (TRADD KO).

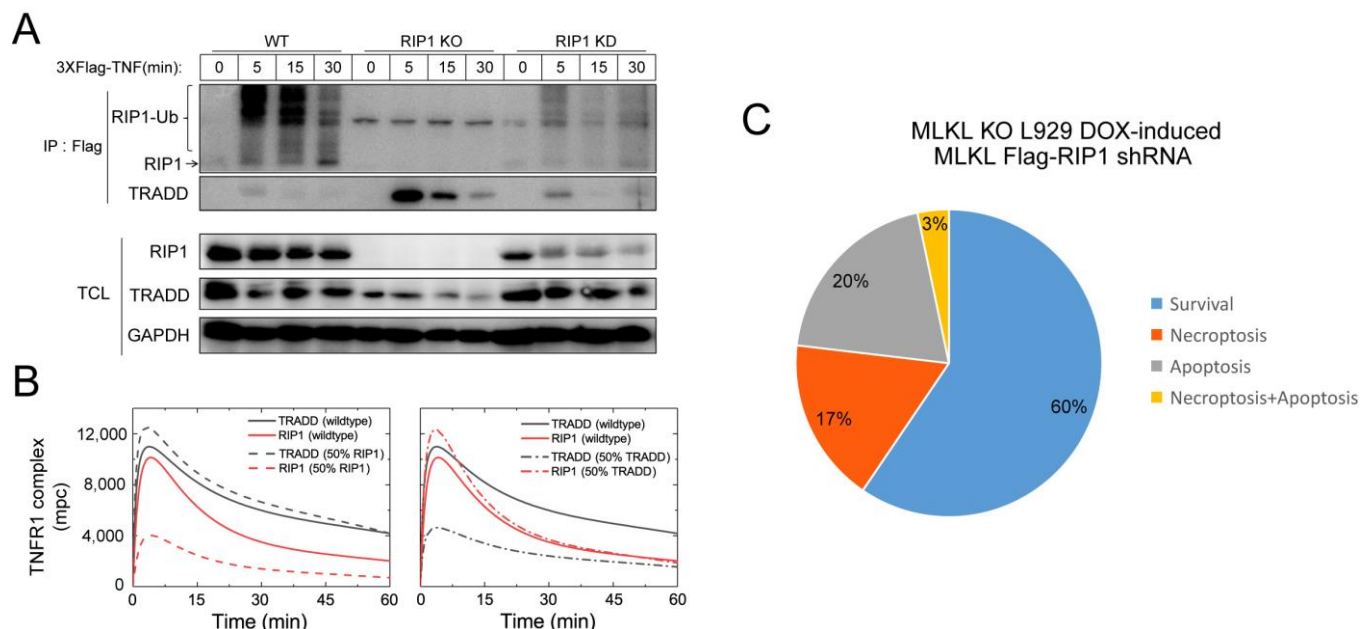

Figure S6. Cell death modes controlled by RIP1 level.

(A) RIP1 and TRADD compete for being recruited to TNFR1. WT, RIP1 KO and RIP1 KD cells were treated with 3xFlag-TNF for indicated periods of time and then immunoprecipitated with anti-Flag antibodies. Immunocomplexes and cell lysates were analyzed by Western blotting with the indicated antibodies.

(B) Simulated effects of RIP1 decrease (left panel) and TRADD decrease (right panel) on the amounts of RIP1 and TRADD in TNFR1 complex. Solid black and red lines correspond to the amounts of TRADD and RIP1 in TNFR1 complex of WT cells. Dashed lines and dashed-dotted lines are the results with 50% RIP1 and 50% TRADD, respectively.

(C) Quantified ratio of different outcomes of cell death with apoptosis, necroptosis, and apoptosis+necroptosis in individual cells from Fig. 6D.

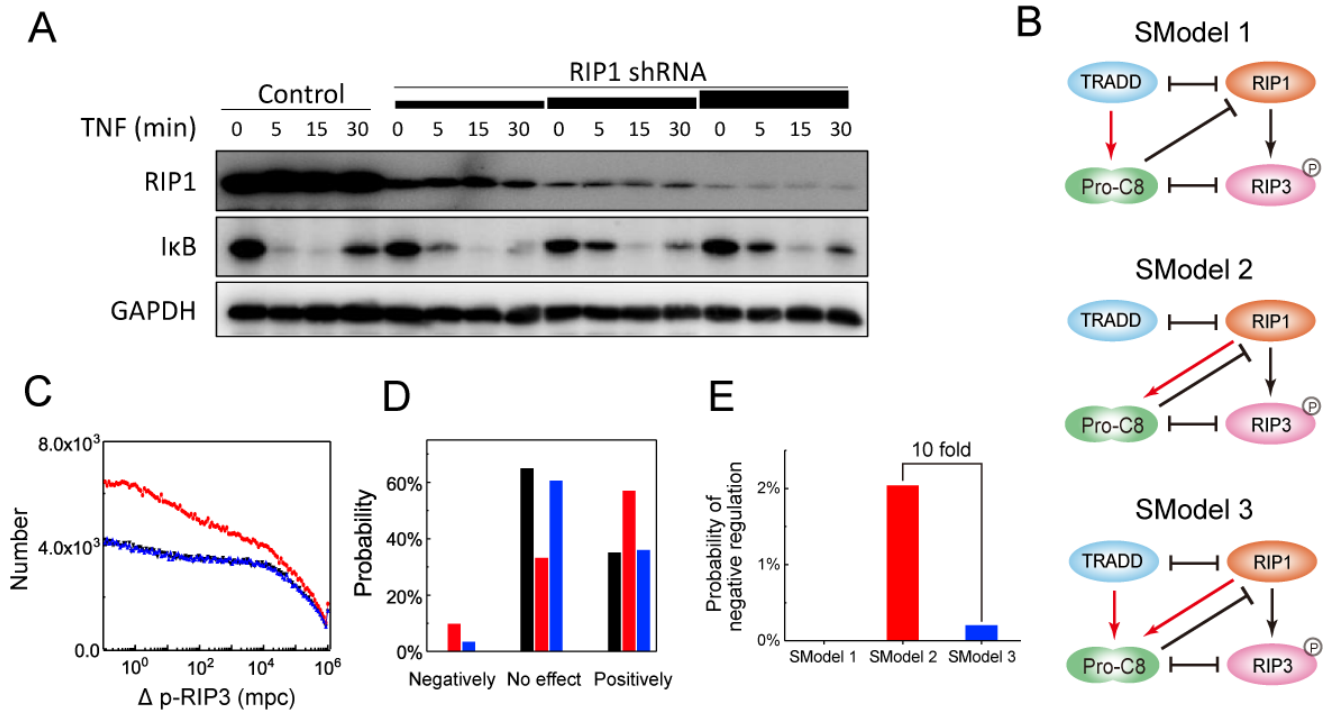

Figure S7. Role of RIP1 in regulating NF- κ B signaling and simplified models with randomly generated parameters.

(A) RIP1 KD barely affects NF- κ B activation. RIP1 was knocked down to different levels and the cells were then treated with TNF for indicated periods of time and were analyzed by Western blotting with the indicated antibodies.

(B) The three simplified models.

(C) Δp -RIP3 set number distributions of RIP1 positively regulates p-RIP3 (Δp -RIP3 > 0) deduced from SModel 1 (black dotted line), SModel 2 (red dotted line) and SModel 3 (blue dotted line) with randomly generated parameters. Each dot represents the parameter set number for each model to achieve the corresponding Δp -RIP3.

(D) Probabilities of different types of p-RIP3 regulation by RIP1 deduced from SModels 1, 2 and 3.

(E) Probabilities of Δp -RIP3 < 0 deduced from simplified models using Gillespie algorithm analysis.

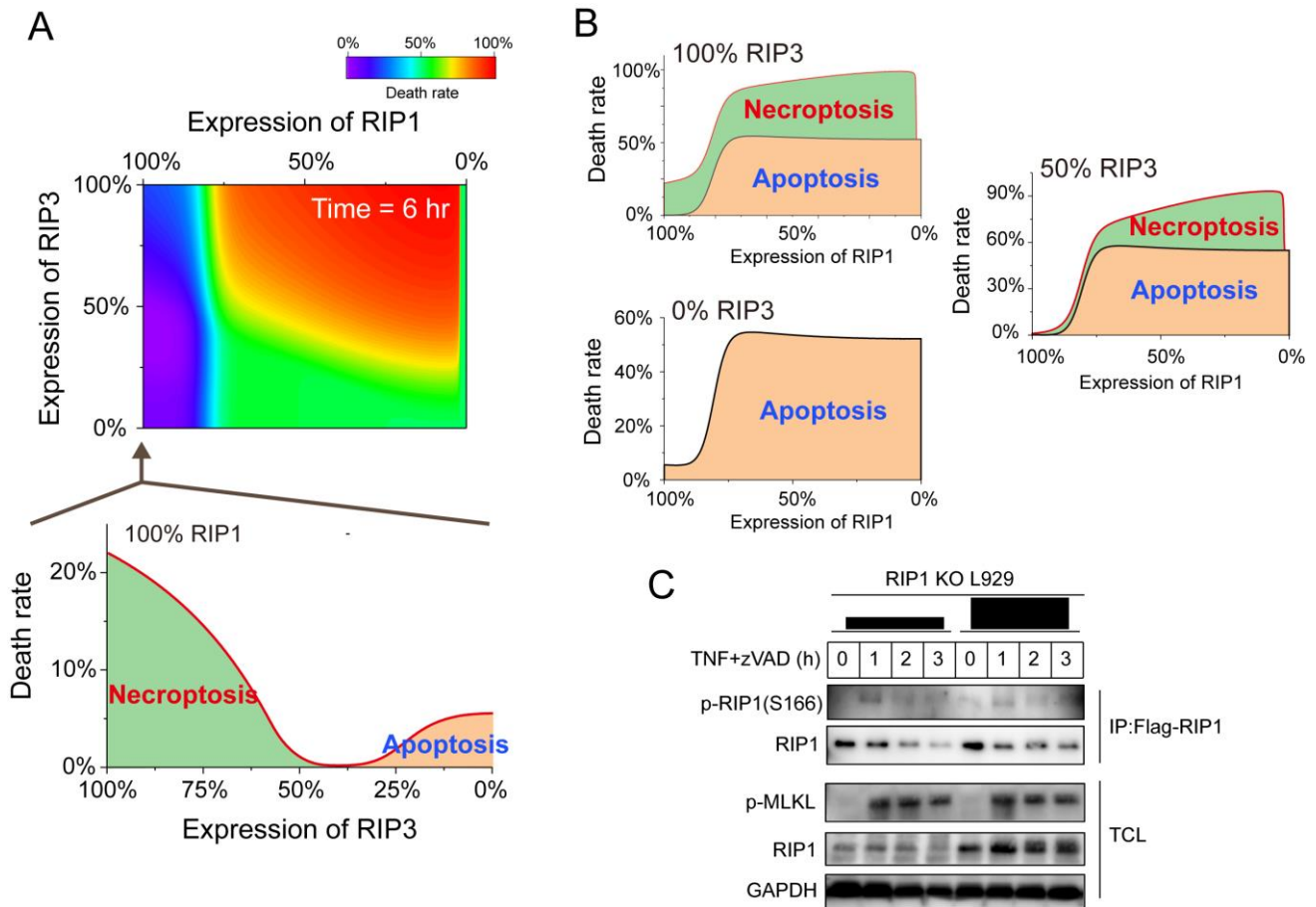

Figure S8. Roles of RIP3 level in regulating cell death mode and the relationship between RIP1 level and RIP1 phosphorylation.

(A) The response of death rate to the decrease of RIP1 under different RIP3 expression levels after 6 hours TNF treatment. Down panel is the contribution proportions of necroptosis and apoptosis to the total cell death under different RIP3 levels when RIP1 level is 100%.

(B) The contribution proportions of necroptosis and apoptosis to the total cell death under different RIP1 levels when RIP3 level is 100%, 50% and 0% respectively after 6 hours TNF treatment.

(C) RIP1 phosphorylation is evaluated by using p-S166 RIP1, which is the biomarker of RIP1 kinase activation (Meng et al., 2018).

Table S1. Product ion areas of Beta-galactosidase and human MLKL peptides in SWATH-MS data. (File can be accessed at <ftp://PASS01429:KS769m@ftp.peptideatlas.org/>.)

Table S2. The peptides included in TNFR1, RIP1 and RIP3 spectral libraries, quantified peptides in TNFR1, RIP1 and RIP3 dataset and manual inspection of the peptide areas. (File can be accessed at <ftp://PASS01429:KS769m@ftp.peptideatlas.org/>.)

Table S3. Absolute quantification of proteins in TNFR1, RIP1 and RIP3 dataset using heavy amino acid labeled peptides. (File can be accessed at <ftp://PASS01429:KS769m@ftp.peptideatlas.org/>.)

Table S4. Reactions and reaction rates of the models.

Table S5. Ordinary differential equations (ODEs) and initial amounts.

Table S6. Parameter values and descriptions of the models.

Table S7. Quantification of Western blotting results using Image J software. (File can be accessed at <ftp://PASS01429:KS769m@ftp.peptideatlas.org/>.)

Table S4. Reactions and reaction rates of the models.

No.	Reactions	Reaction rates
V1	$TNF + TNFR1 \leftrightarrow TNF\_TNFR1$	$k_1^{on} \cdot [TNF] \cdot [TNFR1] - k_1^{off} \cdot [TNF\_TNFR1]$
V2	$TNF\_TNFR1 \rightarrow TNF\_ + TNFR1_{drop}$	$k_2 \cdot [TNF\_TNFR1]$
V3	$TNFR1_{drop} \rightarrow TNFR1$	$k_3 \cdot [TNFR1_{drop}]$
V4	$TNF\_TNFR1 + 2*TRADD \leftrightarrow TNF\_TNFR1\_TRADD_2$	$k_4^{on} \cdot [TNF\_TNFR1] \cdot [TRADD]^2 - k_4^{off} \cdot [TNF\_TNFR1\_TRADD_2]$
V5	$TNF\_TNFR1\_TRADD_2 \rightarrow TNF\_TNFR1_T + 2*TRADD_{drop}$	$k_5 \cdot [TNF\_TNFR1\_TRADD_2]$
V6	$TRADD_{drop} \rightarrow TRADD$	$k_6 \cdot [TRADD_{drop}]$
V7	$TNF\_TNFR1_T \rightarrow TNF\_ + TNFR1_{drop}$	$k_7 \cdot [TNF\_TNFR1_T]$
V8	$TNF\_TNFR1 + 2*RIP1 \leftrightarrow TNF\_TNFR1\_RIP1_2$	$k_8^{on} \cdot [TNF\_TNFR1] \cdot [RIP1]^2 - k_8^{off} \cdot [TNF\_TNFR1\_RIP1_2]$
V9	$TNF\_TNFR1\_RIP1_2 \rightarrow TNF\_TNFR1_R + 2*RIP1_{drop}$	$k_9 \cdot [TNF\_TNFR1\_RIP1_2]$
V10	$RIP1_{drop} \rightarrow RIP1$	$k_{10} \cdot [RIP1_{drop}]$
V11	$TNF\_TNFR1_R \rightarrow TNF\_ + TNFR1_{drop}$	$k_{11} \cdot [TNF\_TNFR1_R]$
V12	$RIP1_{drop} + FADD \leftrightarrow RIP1_{drop\_FADD}$	$k_{12}^{on} \cdot [RIP1_{drop}] \cdot [FADD] - k_{12}^{off} \cdot [RIP1_{drop\_FADD}]$
V13	$RIP1_{drop\_FADD} \rightarrow RIP1_{drop} + FADD_{bind}$	$k_{13} \cdot [RIP1_{drop\_FADD}]$
V14	$FADD_{bind} + pC8 \rightarrow FADD_{bind} + pC8_{bind}$	$k_{14} \cdot [pC8] \cdot [FADD_{bind}]$
V15	$pC8_{bind} \rightarrow C8_{RIP1}$	$k_{15} \cdot [pC8_{bind}]^{n_{15}} / ([pC8_{bind}]^{n_{15}} + K_{pC8}^{n_{15}}) \cdot [FADD_{bind}] / ([FADD_{bind}] + K_{C8R}) \cdot ([pC8_{tot}] - [C8_{RIP1}] - [C8_{RIP1\_RIP3_{pho}}] - [C8_{RIP1\_Bar}] - [pC8_{bind\_RIP3_{bind}}])$
V16	$RIP1_{drop} + RIP3 \rightarrow RIP1_{drop} + RIP3_{bind}$	$k_{16} \cdot [RIP3] \cdot [RIP1_{drop}]^{n_{16}} / ([RIP1_{drop}]^{n_{16}} + K_{RIP1}^{n_{16}})$
V17	$RIP3_{bind} \rightarrow RIP3_{pho}$	$k_{17} \cdot [RIP3_{bind}]^{n_{17}} / ([RIP3_{bind}]^{n_{17}} + K_{RIP3}^{n_{17}}) \cdot ([RIP3_{tot}] - [RIP3_{pho}] - [C8_{RIP1\_RIP3_{pho}}] - [pC8_{bind\_RIP3_{bind}}])$
V18	$pC8_{bind} + RIP3_{bind} \leftrightarrow pC8_{bind\_RIP3_{bind}}$	$k_{18}^{on} \cdot [pC8_{bind}] \cdot [RIP3_{bind}] - k_{18}^{off} \cdot [pC8_{bind\_RIP3_{bind}}]$
V19	$C8_{RIP1} + RIP3_{pho} \leftrightarrow C8_{RIP1\_RIP3_{pho}}$	$k_{19}^{on} \cdot [C8_{RIP1}] \cdot [RIP3_{pho}] - k_{19}^{off} \cdot [C8_{RIP1\_RIP3_{pho}}]$
V20	$C8_{RIP1} + Bar \leftrightarrow C8_{RIP1\_Bar}$	$k_{20}^{on} \cdot [C8_{RIP1}] \cdot [Bar] - k_{20}^{off} \cdot [C8_{RIP1\_Bar}]$
V21	$RIP1_{drop} + RIP3_{bind} \rightarrow RIP1_{bind} + RIP3_{bind}$	$k_{21} \cdot [RIP3_{bind}] / ([RIP3_{bind}] + K_{RIP3-1})$
V22	$Ppm1b + RIP3_{bind} \leftrightarrow Ppm1b_{bind} + RIP3_{bind}$	$k_{22} \cdot [Ppm1b] \cdot [RIP3_{bind}]^{n_{22}} / ([RIP3_{bind}]^{n_{22}} + K_{Ppm1b}^{n_{22}})$
V23	$Ppm1b_{bind} + RIP3_{bind} \leftrightarrow Ppm1b\_RIP3_{bind}$	$k_{23}^{on} \cdot [RIP3_{bind}] \cdot [Ppm1b_{bind}]^{n_{23}} / ([Ppm1b_{bind}]^{n_{23}} + K_{Ppm1b_{bind}}^{n_{23}}) \cdot (Ppm1b_{tot} - Ppm1b\_RIP3_{bind}) - k_{23}^{off} \cdot [Ppm1b\_RIP3_{bind}]$
V24	$RIP3_{pho} + MLKL \leftrightarrow RIP3_{pho\_MLKL}$	$k_{24}^{on} \cdot [RIP3_{pho}] \cdot [MLKL] - k_{24}^{off} \cdot [RIP3_{pho\_MLKL}]$
V25	$RIP3_{pho\_MLKL} \rightarrow RIP3_{pho} + MLKL_{bind}$	$k_{25} \cdot [RIP3_{pho\_MLKL}]$
V26	$MLKL_{bind} \leftrightarrow MLKL_{pho}$	$k_{26}^{on} \cdot [MLKL_{bind}] \cdot ([MLKL_{tot}] - [MLKL_{pho}]) - k_{26}^{off} \cdot [MLKL_{pho}]$
V27	$C8_{RIP1} + pC3 \rightarrow C8_{RIP1} + C3$	$k_{27} \cdot [C8_{RIP1}]^{n_{27}} / ([C8_{RIP1}]^{n_{27}} + K_{C8}^{n_{27}}) \cdot [pC3]$
V28	$TRADD_{drop} + FADD \rightarrow TRADD_{drop} + FADD_{bindT}$	$k_{28} \cdot [FADD] \cdot [TRADD_{drop}]^{n_{28}} / ([TRADD_{drop}]^{n_{28}} + K_{FADDT}^{n_{28}})$
V29	$FADD_{bindT} + pC8 \rightarrow FADD_{bindT} + pC8_{bindT}$	$k_{29} \cdot [pC8] \cdot [FADD_{bindT}]$
V30	$pC8_{bindT} \rightarrow C8_{TRADD}$	$k_{30} \cdot [pC8_{bindT}]^{n_{30}} / ([pC8_{bindT}]^{n_{30}} + K_{pC8_T}^{n_{30}}) \cdot [FADD_{bindT}] / ([FADD_{bindT}] + K_{C8T}) \cdot [pC8]$
V31	$C8_{TRADD} + pC3 \rightarrow C8_{TRADD} + C3$	$k_{31} \cdot [C8_{TRADD}]^{n_{31}} / ([C8_{TRADD}]^{n_{31}} + K_{C8}^{n_{31}}) \cdot [pC3]$
V32	$pC8_{bindT} + RIP3_{bind} \leftrightarrow pC8_{bindT\_RIP3_{bind}}$	$k_{32}^{on} \cdot [pC8_{bindT}] \cdot [RIP3_{bind}] - k_{32}^{off} \cdot [pC8_{bindT\_RIP3_{bind}}]$
V33	$C8_{TRADD} + RIP3_{pho} \leftrightarrow C8_{TRADD\_RIP3_{pho}}$	$k_{33}^{on} \cdot [C8_{TRADD}] \cdot [RIP3_{pho}] - k_{33}^{off} \cdot [C8_{TRADD\_RIP3_{pho}}]$
V34	$pC8_{bind} + RIP1_{bind} \leftrightarrow pC8_{bind}$	$k_{34} \cdot [pC8_{bind}] \cdot [RIP1_{bind}]$
V35	$pC8_{bindT} + RIP1_{bind} \leftrightarrow pC8_{bindT}$	$k_{35} \cdot [pC8_{bindT}] \cdot [RIP1_{bind}]$
V36	$RIP1_{drop} \rightarrow \emptyset$	$k_{36} \cdot [RIP1_{drop}]$
V37	$FADD_{bind} \rightarrow \emptyset$	$k_{37} \cdot [FADD_{bind}]$
V38	$pC8_{bind} \rightarrow \emptyset$	$k_{38} \cdot [pC8_{bind}]$
V39	$pC8_{bind\_RIP3_{bind}} \rightarrow \emptyset$	$k_{39} \cdot [pC8_{bind\_RIP3_{bind}}]$
V40	$Ppm1b_{bind} \rightarrow \emptyset$	$k_{40} \cdot [Ppm1b_{bind}]$

V41	$\text{RIP3}_{\text{bind}} \rightarrow \emptyset$	$k_{41} \cdot [\text{RIP3}_{\text{bind}}]$
V42	$\text{RIP1}_{\text{bind}} \rightarrow \emptyset$	$k_{42} \cdot [\text{RIP1}_{\text{bind}}]$
V43	$\text{FADD}_{\text{bindT}} \rightarrow \emptyset$	$k_{43} \cdot [\text{FADD}_{\text{bindT}}]$
V44	$\text{pC8}_{\text{bindT}} \rightarrow \emptyset$	$k_{44} \cdot [\text{pC8}_{\text{bindT}}]$
V45	$\text{C8}_{\text{TRADD}} \rightarrow \emptyset$	$k_{45} \cdot [\text{C8}_{\text{TRADD}}]$
V46	$\text{C3} \rightarrow \emptyset$	$k_{46} \cdot [\text{C3}]$

Model 1 includes above reactions with white and light-green background. Model 2 involves above reactions with white and light-blue background. All above reactions are contained in Model 3. Model 4 is also described by all above reactions apart from Reactions V32, V33 and V35.

Table S5. Ordinary differential equations (ODEs) and initial amounts.

No.	Components	ODEs	Initial amounts (mpc)	References
E1	TNF	$d[\text{TNF}]/dt = -V1$	3600	Measured
E2	TNFR1	$d[\text{TNFR1}]/dt = -V1 + V3$	20000	Estimated
E3	TNF_TNFR1	$d[\text{TNF\_TNFR1}]/dt = V1 - V2 - V4 - V8$	0	
E4	TNF_	$d[\text{TNF\_}]/dt = V2 + V7 + V11$	0	
E5	TNFR1 _{drop}	$d[\text{TNFR1}_{\text{drop}}]/dt = V2 - V3 + V7 + V11$	0	
E6	TRADD	$d[\text{TRADD}]/dt = -2*V4 + V6$	20000	(Schwanhäusser et al., 2011)
E7	TNF_TNFR1_TRADD ₂	$d[\text{TNF\_TNFR1\_TRADD}_2]/dt = V4 - V5$	0	
E8	TNF_TNFR1 _T	$d[\text{TNF\_TNFR1}_T]/dt = V5 - V7$	0	
E9	TRADD _{drop}	$d[\text{TRADD}_{\text{drop}}]/dt = 2*V5 - V6$	0	
E10	RIP1	$d[\text{RIP1}]/dt = -2*V8 + V10$	51000	Measured
E11	TNF_TNFR1_RIP1 ₂	$d[\text{TNF\_TNFR1\_RIP1}_2]/dt = V8 - V9$	0	
E12	TNF_TNFR1 _R	$d[\text{TNF\_TNFR1}_R]/dt = V9 - V11$	0	
E13	RIP1 _{drop}	$d[\text{RIP1}_{\text{drop}}]/dt = 2*V9 - V10 - V12 + V13 - V36$	0	
E14	FADD	$d[\text{FADD}]/dt = -V12 - V28$	24000	(Schwanhäusser et al., 2011)
E15	RIP1 _{drop} _FADD	$d[\text{RIP1}_{\text{drop\_FADD}}]/dt = V12 - V13$	0	
E16	FADD _{bind}	$d[\text{FADD}_{\text{bind}}]/dt = V13 - V37$	0	
E17	pC8	$d[\text{pC8}]/dt = -V14 - V15$	25000	Estimated
E18	pC8 _{bind}	$d[\text{pC8}_{\text{bind}}]/dt = V14 - V18 - V38$		
E19	C8 _{RIP1}	$d[\text{C8}_{\text{RIP1}}]/dt = V15 - V19 - V20$	0	
E20	RIP3	$d[\text{RIP3}]/dt = -V16$	32000	Measured
E21	RIP3 _{bind}	$d[\text{RIP3}_{\text{bind}}]/dt = V16 - V18 - V23 - V32 - V41$	0	
E22	RIP3 _{pho}	$d[\text{RIP3}_{\text{pho}}]/dt = V17 - V19 - V24 + V25 - V33$	0	
E23	pC8 _{bind} _RIP3 _{bind}	$d[\text{pC8}_{\text{bind\_RIP3}_{\text{bind}}}] / dt = V18 - V39$	0	
E24	C8 _{RIP1} _RIP3 _{pho}	$d[\text{C8}_{\text{RIP1\_RIP3}_{\text{pho}}}] / dt = V19$	0	
E25	Bar	$d[\text{Bar}]/dt = -V20$	1000	(Schwanhäusser et al., 2011)
E26	C8 _{RIP1} _Bar	$d[\text{C8}_{\text{RIP1\_Bar}}]/dt = V20$	0	
E27	RIP1 _{bind}	$d[\text{RIP1}_{\text{bind}}]/dt = V21 - V42 - V34 - V35$	0	
E28	Ppm1b	$d[\text{Ppm1b}]/dt = -V22 - V23$	20450	Estimated
E29	Ppm1b _{bind}	$d[\text{Ppm1b}_{\text{bind}}]/dt = V22 - V40$	0	
E30	Ppm1b_RIP3 _{bind}	$d[\text{Ppm1b\_RIP3}_{\text{bind}}]/dt = V23$	0	
E31	MLKL	$d[\text{MLKL}]/dt = -V24$	15000	Estimated
E32	RIP3 _{pho} _MLKL	$d[\text{RIP3}_{\text{pho\_MLKL}}]/dt = V24 - V25$	0	
E33	MLKL _{bind}	$d[\text{MLKL}_{\text{bind}}]/dt = V25$	0	
E34	MLKL _{pho}	$d[\text{MLKL}_{\text{pho}}]/dt = V26$	0	
E35	FADD _{bindT}	$d[\text{FADD}_{\text{bindT}}]/dt = V28 - V43$	0	
E36	pC8 _{bindT}	$d[\text{pC8}_{\text{bindT}}]/dt = V29 - V32 - V44$	0	
E37	C8 _{TRADD}	$d[\text{C8}_{\text{TRADD}}]/dt = V30 - V33 - V45$	0	
E38	pC8 _{bindT} _RIP3 _{bind}	$d[\text{pC8}_{\text{bindT\_RIP3}_{\text{bind}}}] / dt = V32$	0	
E39	C8 _{TRADD} _RIP3 _{pho}	$d[\text{C8}_{\text{TRADD\_RIP3}_{\text{pho}}}] / dt = V33$	0	
E40	pC3	$d[\text{pC3}]/dt = -V27 - V31$	97000	(Schwanhäusser et al., 2011)
E41	C3	$d[\text{C3}]/dt = V27 + V31 - V46$	0	

Model 1 includes above reaction rates in ODEs with white and light-green background. Model 2 involves above reaction rates with white and light-blue background. All above reactions are contained in Model 3. Model 4 is also described by all above reaction rates in ODEs apart from reaction rate of V32, V33 and V35.

Reference

B. Schwanhaeusser, D. Busse, N. Li, G. Dittmar, J. Schuchhardt, J. Wolf, W. Chen, M. Selbach, Global quantification of mammalian gene expression control. *Nature* **473**, 337-342 (2011).

Table S6. Parameters values and descriptions of the models.

Parameters in Model 1

Parameters	Description	References
$k_1^{\text{on}} = 3.83 \times 10^{-7} \text{ mpc}^{-1} \cdot \text{s}^{-1}$, $k_1^{\text{off}} = 1.0 \times 10^{-3} \text{ s}^{-1}$	Rates of TNF binds to TNFR1	Fitted and (Albeck et al., 2008)
$k_2 = 4.1 \times 10^{-1} \text{ s}^{-1}$	Rate of TNFR1 drops from TNF_TNFR1 complex	Fitted
$k_3 = 4.78 \times 10^{-4} \text{ s}^{-1}$	Inactivation rate of the dropped TNFR1	Fitted
$k_4^{\text{on}} = 3.07 \times 10^{-9} \text{ mpc}^{-2} \cdot \text{s}^{-1}$, $k_4^{\text{off}} = 1.0 \times 10^{-3} \text{ s}^{-1}$	Rate of TNF_TNFR1 complex recruits TRADD	Fitted and (Albeck et al., 2008)
$k_5 = 2.29 \times 10^{-4} \text{ s}^{-1}$	TRADD disassociation rate from Complex I	Fitted
$k_6 = 1.0 \times 10^{-7} \text{ s}^{-1}$	Inactivation rate of the dropped TRADD	Estimated
$k_7 = 5.19 \times 10^{-4} \text{ s}^{-1}$	Rate of TNFR1 drops from TNF_TNFR_TRADD complex	Fitted
$k_8^{\text{on}} = 2.38 \times 10^{-10} \text{ mpc}^{-2} \cdot \text{s}^{-1}$, $k_8^{\text{off}} = 1.0 \times 10^{-3} \text{ s}^{-1}$	Rate of TNF_TNFR1 complex recruits RIP1	Fitted and (Albeck et al., 2008)
$k_9 = 9.69 \times 10^{-4} \text{ s}^{-1}$	RIP1 disassociation rate from Complex I	Fitted
$k_{10} = 1.0 \times 10^{-7} \text{ s}^{-1}$	Inactivation rate of the dropped RIP1	Estimated
$k_{11} = 5.33 \times 10^{-4} \text{ s}^{-1}$	Rate of TNFR1 drops from TNF_TNFR_RIP1 complex	Fitted
$k_{16} = 8.0 \times 10^{-5} \text{ s}^{-1}$, $K_{\text{RIP1}} = 1.0 \text{ mpc}$, $n_{16} = 15$	Rate and michaelis constant of RIP3 binds to RIP1	Fitted
$k_{17} = 6.84 \times 10^{-5} \text{ s}^{-1}$, $K_{\text{RIP3}} = 16 \text{ mpc}$, $n_{17} = 2$	Rate and michaelis constant of RIP3 phosphorylation	Estimated
$k_{21} = 5.48 \times 10^{-2} \text{ mpc} \cdot \text{s}^{-1}$, $K_{\text{RIP3-1}} = 100 \text{ mpc}$	Rate and michaelis constant of RIP1 binds to RIP3	Fitted
$k_{22} = 1.4 \times 10^{-5} \text{ mpc} \cdot \text{s}^{-1}$, $K_{\text{Ppm1b}} = 10 \text{ mpc}$, $n_{22} = 4$	Rate and michaelis constant of Ppm1b binds to RIP3	Fitted
$k_{23}^{\text{on}} = 9.66 \times 10^{-4} \text{ mpc}^{-1} \cdot \text{s}^{-1}$, $K_{\text{Ppm1b}_{\text{bind}}} = 10^{-2} \text{ mpc}$, $n_{23} = 4$, $k_{23}^{\text{off}} = 3.53 \times 10^{-2} \text{ s}^{-1}$	Rates of negative effect of Ppm1b on RIP3	Estimated
$k_{24}^{\text{on}} = 4.5 \times 10^{-10} \text{ mpc}^{-1} \cdot \text{s}^{-1}$, $k_{24}^{\text{off}} = 1.0 \times 10^{-3} \text{ s}^{-1}$	Rates of MLKL binds to RIP3	Fitted and (Albeck et al., 2008)
$k_{25} = 1.0 \text{ s}^{-1}$	Activation rate of MLKL	(Albeck et al., 2008)
$k_{26}^{\text{on}} = 1.5 \times 10^{-7} \text{ mpc}^{-1} \cdot \text{s}^{-1}$, $k_{26}^{\text{off}} = 1.0 \times 10^{-4} \text{ s}^{-1}$	Phosphorylation rates of MLKL	Estimated
$k_{28} = 1.0 \times 10^{-4} \text{ s}^{-1}$, $K_{\text{FADDT}} = 2.5 \times 10^3 \text{ mpc}$, $n_{28} = 20$	Rates of TRADD binds to FADD	Estimated
$k_{29} = 1.0 \times 10^{-6} \text{ mpc}^{-1} \cdot \text{s}^{-1}$	Rates of Pro-caspase-8 binds to FADD	Estimated
$k_{30} = 1.6 \times 10^{-3} \text{ s}^{-1}$, $K_{\text{pC8}_t} = 10^{-2} \text{ mpc}$, $n_{30} = 4$, $K_{\text{C8T}} = 10 \text{ mpc}$	Rate and michaelis constant of Caspase-8 activation	Estimated
$k_{31} = 2.0 \times 10^{-4} \text{ s}^{-1}$, $K_{\text{C8}} = 1.0 \times 10^4 \text{ mpc}$, $n_{31} = 4$	Rate and michaelis constant of Caspase-3 activation	Estimated
$k_{32}^{\text{on}} = 1.0 \times 10^{-2} \text{ mpc}^{-1} \cdot \text{s}^{-1}$, $k_{32}^{\text{off}} = 1.0 \times 10^{-2} \text{ s}^{-1}$	Rates of Pro-caspase-8 inhibits RIP3	(Albeck et al., 2008)
$k_{33}^{\text{on}} = 1.0 \times 10^{-2} \text{ mpc}^{-1} \cdot \text{s}^{-1}$, $k_{33}^{\text{off}} = 1.0 \times 10^{-2} \text{ s}^{-1}$	Rates of Caspase-8 inhibits p-RIP3	(Albeck et al., 2008)
$k_{35} = 1.0 \times 10^{-6} \text{ mpc}^{-1} \cdot \text{s}^{-1}$	Rates of Pro-caspase-8 inhibits RIP1	Fitted
$k_{36} = 1.0 \times 10^{-4} \text{ s}^{-1}$	Degradation rate of dropped RIP1	Fitted
$k_{40} = 5.5 \times 10^{-5} \text{ s}^{-1}$	Degradation rate of Ppm1b that binds to RIP3	Fitted

$k_{41} = 1.8 \times 10^{-3} \text{ s}^{-1}$	Degradation rate of RIP3 that binds to RIP1	Fitted
$k_{42} = 9.49 \times 10^{-5} \text{ s}^{-1}$	Degradation rate of RIP1 that binds to RIP3	Fitted
$k_{43} = 1.0 \times 10^{-4} \text{ s}^{-1}$	Degradation rate of FADD that binds to TRADD	Estimated
$k_{44} = 1.0 \times 10^{-3} \text{ s}^{-1}$	Degradation rate of Pro-caspase-8 that binds to FADD	Estimated
$k_{45} = 6.0 \times 10^{-4} \text{ s}^{-1}$	Degradation rate of Caspase-8 that activated by TRADD	Estimated
$k_{46} = 3.0 \times 10^{-5} \text{ s}^{-1}$	Degradation rate of Caspase-3	Estimated

Parameters in Model 2

Parameters	Description	References
$k_1^{\text{on}} = 3.83 \times 10^{-7} \text{ mpc}^{-1} \cdot \text{s}^{-1}$, $k_1^{\text{off}} = 1.0 \times 10^{-3} \text{ s}^{-1}$	Rates of TNF binds to TNFR1	Fitted and (Albeck et al., 2008)
$k_2 = 4.1 \times 10^{-1} \text{ s}^{-1}$	Rate of TNFR1 drops from TNF_TNFR1 complex	Fitted
$k_3 = 4.78 \times 10^{-4} \text{ s}^{-1}$	Inactivation rate of the dropped TNFR1	Fitted
$k_4^{\text{on}} = 3.07 \times 10^{-9} \text{ mpc}^{-2} \cdot \text{s}^{-1}$, $k_4^{\text{off}} = 1.0 \times 10^{-3} \text{ s}^{-1}$	Rate of TNF_TNFR1 complex recruits TRADD	Fitted and (Albeck et al., 2008)
$k_5 = 2.29 \times 10^{-4} \text{ s}^{-1}$	TRADD disassociation rate from Complex I	Fitted
$k_6 = 1.0 \times 10^{-7} \text{ s}^{-1}$	Inactivation rate of the dropped TRADD	Estimated
$k_7 = 5.19 \times 10^{-4} \text{ s}^{-1}$	Rate of TNFR1 drops from TNF_TNFR_TRADD complex	Fitted
$k_8^{\text{on}} = 2.38 \times 10^{-10} \text{ mpc}^{-2} \cdot \text{s}^{-1}$, $k_8^{\text{off}} = 1.0 \times 10^{-3} \text{ s}^{-1}$	Rate of TNF_TNFR1 complex recruits RIP1	Fitted and (Albeck et al., 2008)
$k_9 = 9.69 \times 10^{-4} \text{ s}^{-1}$	RIP1 disassociation rate from Complex I	Fitted
$k_{10} = 1.0 \times 10^{-7} \text{ s}^{-1}$	Inactivation rate of the dropped RIP1	Estimated
$k_{11} = 5.33 \times 10^{-4} \text{ s}^{-1}$	Rate of TNFR1 drops from TNF_TNFR_RIP1 complex	Fitted
$k_{12}^{\text{on}} = 7.0 \times 10^{-10} \text{ mpc}^{-1} \cdot \text{s}^{-1}$, $k_{12}^{\text{off}} = 1.0 \times 10^{-3} \text{ s}^{-1}$	Rates of RIP1 binds to FADD	Fitted and (Albeck et al., 2008)
$k_{13} = 1.0 \text{ s}^{-1}$	Activation rate of FADD	(Albeck et al., 2008)
$k_{14} = 1.0 \times 10^{-8} \text{ mpc}^{-1} \cdot \text{s}^{-1}$	Rates of Pro-caspase-8 binds to FADD	Estimated
$k_{15} = 9.89 \times 10^{-4} \text{ s}^{-1}$, $n_{15} = 4$,<br>$K_{\text{pc8}} = 10^{-2} \text{ mpc}$, $K_{\text{c8R}} = 1.83 \times 10^3 \text{ mpc}$	Rate and michaelis constant of Caspase-8 activation	Estimated
$k_{16} = 8.0 \times 10^{-5} \text{ s}^{-1}$, $K_{\text{RIP1}} = 1.0 \text{ mpc}$, $n_{16} = 15$	Rate and michaelis constant of RIP3 binds to RIP1	Fitted
$k_{17} = 6.84 \times 10^{-5} \text{ s}^{-1}$, $K_{\text{RIP3}} = 16 \text{ mpc}$, $n_{17} = 2$	Rate and michaelis constant of RIP3 phosphorylation	Estimated
$k_{18}^{\text{on}} = 1.0 \times 10^{-2} \text{ mpc}^{-1} \cdot \text{s}^{-1}$, $k_{18}^{\text{off}} = 1.0 \times 10^{-2} \text{ s}^{-1}$	Rates of Pro-caspase-8 inhibits RIP3	(Albeck et al., 2008)
$k_{19}^{\text{on}} = 1.0 \times 10^{-2} \text{ mpc}^{-1} \cdot \text{s}^{-1}$, $k_{19}^{\text{off}} = 1.0 \times 10^{-2} \text{ s}^{-1}$	Rates of Caspase-8 inhibits p-RIP3	(Albeck et al., 2008)
$k_{20}^{\text{on}} = 1.0 \times 10^{-2} \text{ mpc}^{-1} \cdot \text{s}^{-1}$, $k_{20}^{\text{off}} = 1.0 \times 10^{-2} \text{ s}^{-1}$	Rates of Bar inhibits Caspase-8	(Albeck et al., 2008)
$k_{21} = 4.0 \times 10^{-2} \text{ mpc} \cdot \text{s}^{-1}$, $K_{\text{RIP3-1}} = 100 \text{ mpc}$	Rate and michaelis constant of RIP1 binds to RIP3	Fitted
$k_{22} = 1.4 \times 10^{-5} \text{ mpc} \cdot \text{s}^{-1}$, $K_{\text{ppm1b}} = 10 \text{ mpc}$, $n_{22} = 4$	Rate and michaelis constant of Ppm1b binds to RIP3	Fitted

$k_{23}^{on} = 9.66 \times 10^{-4} \text{ mpc}^{-1} \cdot \text{s}^{-1}$, $K_{\text{Ppm1b bind}} = 10^{-2} \text{ mpc}$,<br>$n_{23} = 4, k_{23}^{off} = 3.53 \times 10^{-2} \text{ s}^{-1}$	Rates of negative effect of Ppm1b on RIP3	Estimated
$k_{24}^{on} = 4.5 \times 10^{-10} \text{ mpc}^{-1} \cdot \text{s}^{-1}$, $k_{24}^{off} = 1.0 \times 10^{-3} \text{ s}^{-1}$	Rates of MLKL binds to RIP3	Fitted and<br>(Albeck et al., 2008)
$k_{25} = 1.0 \text{ s}^{-1}$	Activation rate of MLKL	(Albeck et al., 2008)
$k_{26}^{on} = 1.5 \times 10^{-7} \text{ mpc}^{-1} \cdot \text{s}^{-1}$, $k_{26}^{off} = 1.0 \times 10^{-4} \text{ s}^{-1}$	Phosphorylation rates of MLKL	Estimated
$k_{27} = 1.0 \times 10^{-5} \text{ s}^{-1}$, $K_{C8} = 1.0 \times 10^4 \text{ mpc}$, $n_{27} = 4$	Rate and michaelis constant of Caspase-3 activation	Estimated
$k_{34} = 1.0 \times 10^{-6} \text{ mpc}^{-1} \cdot \text{s}^{-1}$	Rates of Pro-caspase-8 inhibits RIP1	Fitted
$k_{36} = 1.0 \times 10^{-4} \text{ s}^{-1}$	Degradation rate of dropped RIP1	Fitted
$k_{37} = 8.0 \times 10^{-5} \text{ s}^{-1}$	Degradation rate of FADD that binds to RIP1	Fitted
$k_{38} = 1.0 \times 10^{-4} \text{ s}^{-1}$	Degradation rate of Pro-caspase-8 that binds to FADD	Estimated
$k_{39} = 1.7 \times 10^{-4} \text{ s}^{-1}$	Degradation rate of Pro-caspase-8 that binds to RIP3	Fitted
$k_{40} = 5.5 \times 10^{-5} \text{ s}^{-1}$	Degradation rate of Ppm1b that binds to RIP3	Fitted
$k_{41} = 1.8 \times 10^{-3} \text{ s}^{-1}$	Degradation rate of RIP3 that binds to RIP1	Fitted
$k_{42} = 8.0 \times 10^{-5} \text{ s}^{-1}$	Degradation rate of RIP1 that binds to RIP3	Fitted
$k_{46} = 3.0 \times 10^{-5} \text{ s}^{-1}$	Degradation rate of Caspase-3	Estimated

Parameters in Model 3

Parameters	Description	References
$k_1^{on} = 3.83 \times 10^{-7} \text{ mpc}^{-1} \cdot \text{s}^{-1}$, $k_1^{off} = 1.0 \times 10^{-3} \text{ s}^{-1}$	Rates of TNF binds to TNFR1	Fitted and<br>(Albeck et al., 2008)
$k_2 = 4.1 \times 10^{-1} \text{ s}^{-1}$	Rate of TNFR1 drops from TNF_TNFR1 complex	Fitted
$k_3 = 4.78 \times 10^{-4} \text{ s}^{-1}$	Inactivation rate of the dropped TNFR1	Fitted
$k_4^{on} = 3.07 \times 10^{-9} \text{ mpc}^{-2} \cdot \text{s}^{-1}$, $k_4^{off} = 1.0 \times 10^{-3} \text{ s}^{-1}$	Rate of TNF_TNFR1 complex recruits TRADD	Fitted and<br>(Albeck et al., 2008)
$k_5 = 2.29 \times 10^{-4} \text{ s}^{-1}$	TRADD disassociation rate from Complex I	Fitted
$k_6 = 1.0 \times 10^{-7} \text{ s}^{-1}$	Inactivation rate of the dropped TRADD	Estimated
$k_7 = 5.19 \times 10^{-4} \text{ s}^{-1}$	Rate of TNFR1 drops from TNF_TNFR_TRADD complex	Fitted
$k_8^{on} = 2.38 \times 10^{-10} \text{ mpc}^{-2} \cdot \text{s}^{-1}$, $k_8^{off} = 1.0 \times 10^{-3} \text{ s}^{-1}$	Rate of TNF_TNFR1 complex recruits RIP1	Fitted and<br>(Albeck et al., 2008)
$k_9 = 9.69 \times 10^{-4} \text{ s}^{-1}$	RIP1 disassociation rate from Complex I	Fitted
$k_{10} = 1.0 \times 10^{-7} \text{ s}^{-1}$	Inactivation rate of the dropped RIP1	Estimated
$k_{11} = 5.33 \times 10^{-4} \text{ s}^{-1}$	Rate of TNFR1 drops from TNF_TNFR_RIP1 complex	Fitted
$k_{12}^{on} = 7.0 \times 10^{-10} \text{ mpc}^{-1} \cdot \text{s}^{-1}$, $k_{12}^{off} = 1.0 \times 10^{-3} \text{ s}^{-1}$	Rates of RIP1 binds to FADD	Fitted and<br>(Albeck et al., 2008)
$k_{13} = 1.0 \text{ s}^{-1}$	Activation rate of FADD	(Albeck et al., 2008)
$k_{14} = 1.0 \times 10^{-8} \text{ mpc}^{-1} \cdot \text{s}^{-1}$	Rates of Pro-caspase-8 binds to FADD	Estimated

$k_{15} = 9.89 \times 10^{-4} \text{ s}^{-1}$, $n_{15} = 4$,<br>$K_{pC8} = 10^{-2} \text{ mpc}$, $K_{C8R} = 1.83 \times 10^3 \text{ mpc}$	Rate and michaelis constant of Caspase-8 activation	Estimated
$k_{16} = 8.0 \times 10^{-5} \text{ s}^{-1}$, $K_{RIP1} = 1.0 \text{ mpc}$, $n_{16} = 15$	Rate and michaelis constant of RIP3 binds to RIP1	Fitted
$k_{17} = 6.84 \times 10^{-5} \text{ s}^{-1}$, $K_{RIP3} = 16 \text{ mpc}$, $n_{17} = 2$	Rate and michaelis constant of RIP3 phosphorylation	Estimated
$k_{18}^{\text{on}} = 1.0 \times 10^{-2} \text{ mpc}^{-1} \cdot \text{s}^{-1}$, $k_{18}^{\text{off}} = 1.0 \times 10^{-2} \text{ s}^{-1}$	Rates of Pro-caspase-8 inhibits RIP3	(Albeck et al., 2008)
$k_{19}^{\text{on}} = 1.0 \times 10^{-2} \text{ mpc}^{-1} \cdot \text{s}^{-1}$, $k_{19}^{\text{off}} = 1.0 \times 10^{-2} \text{ s}^{-1}$	Rates of Caspase-8 inhibits p-RIP3	(Albeck et al., 2008)
$k_{20}^{\text{on}} = 1.0 \times 10^{-2} \text{ mpc}^{-1} \cdot \text{s}^{-1}$, $k_{20}^{\text{off}} = 1.0 \times 10^{-2} \text{ s}^{-1}$	Rates of Bar inhibits Caspase-8	(Albeck et al., 2008)
$k_{21} = 4.0 \times 10^{-2} \text{ mpc} \cdot \text{s}^{-1}$, $K_{RIP3-1} = 100 \text{ mpc}$	Rate and michaelis constant of RIP1 binds to RIP3	Fitted
$k_{22} = 1.4 \times 10^{-5} \text{ mpc} \cdot \text{s}^{-1}$, $K_{Ppm1b} = 10 \text{ mpc}$, $n_{22} = 4$	Rate and michaelis constant of Ppm1b binds to RIP3	Fitted
$k_{23}^{\text{on}} = 9.66 \times 10^{-4} \text{ mpc}^{-1} \cdot \text{s}^{-1}$, $K_{Ppm1b \text{ bind}} = 10^{-2} \text{ mpc}$,<br>$n_{23} = 4$, $k_{23}^{\text{off}} = 3.53 \times 10^{-2} \text{ s}^{-1}$	Rates of negative effect of Ppm1b on RIP3	Estimated
$k_{24}^{\text{on}} = 4.5 \times 10^{-10} \text{ mpc}^{-1} \cdot \text{s}^{-1}$, $k_{24}^{\text{off}} = 1.0 \times 10^{-3} \text{ s}^{-1}$	Rates of MLKL binds to RIP3	Fitted and<br>(Albeck et al., 2008)
$k_{25} = 1.0 \text{ s}^{-1}$	Activation rate of MLKL	(Albeck et al., 2008)
$k_{26}^{\text{on}} = 1.5 \times 10^{-7} \text{ mpc}^{-1} \cdot \text{s}^{-1}$, $k_{26}^{\text{off}} = 1.0 \times 10^{-4} \text{ s}^{-1}$	Phosphorylation rates of MLKL	Estimated
$k_{27} = 1.0 \times 10^{-5} \text{ s}^{-1}$, $K_{C8} = 1.0 \times 10^4 \text{ mpc}$, $n_{27} = 4$	Rate and michaelis constant of Caspase-3 activation	Estimated
$k_{28} = 1.0 \times 10^{-4} \text{ s}^{-1}$, $K_{FADDT} = 2.5 \times 10^3 \text{ mpc}$, $n_{28} = 20$	Rates of TRADD binds to FADD	Estimated
$k_{29} = 1.0 \times 10^{-6} \text{ mpc}^{-1} \cdot \text{s}^{-1}$	Rates of Pro-caspase-8 binds to FADD	Estimated
$k_{30} = 1.6 \times 10^{-3} \text{ s}^{-1}$, $K_{pC8T} = 10^{-2} \text{ mpc}$,<br>$n_{30} = 4$, $K_{C8T} = 10 \text{ mpc}$	Rate and michaelis constant of Caspase-8 activation	Estimated
$k_{31} = 2.0 \times 10^{-4} \text{ s}^{-1}$, $K_{C8} = 1.0 \times 10^4 \text{ mpc}$, $n_{31} = 4$	Rate and michaelis constant of Caspase-3 activation	Estimated
$k_{32}^{\text{on}} = 1.0 \times 10^{-2} \text{ mpc}^{-1} \cdot \text{s}^{-1}$, $k_{32}^{\text{off}} = 1.0 \times 10^{-2} \text{ s}^{-1}$	Rates of Pro-caspase-8 inhibits RIP3	(Albeck et al., 2008)
$k_{33}^{\text{on}} = 1.0 \times 10^{-2} \text{ mpc}^{-1} \cdot \text{s}^{-1}$, $k_{33}^{\text{off}} = 1.0 \times 10^{-2} \text{ s}^{-1}$	Rates of Caspase-8 inhibits p-RIP3	(Albeck et al., 2008)
$k_{34} = 1.0 \times 10^{-6} \text{ mpc}^{-1} \cdot \text{s}^{-1}$	Rates of Pro-caspase-8 inhibits RIP1	Fitted
$k_{35} = 1.0 \times 10^{-6} \text{ mpc}^{-1} \cdot \text{s}^{-1}$	Rates of Pro-caspase-8 inhibits RIP1	Fitted
$k_{36} = 1.0 \times 10^{-4} \text{ s}^{-1}$	Degradation rate of dropped RIP1	Fitted
$k_{37} = 8.0 \times 10^{-5} \text{ s}^{-1}$	Degradation rate of FADD that binds to RIP1	Fitted
$k_{38} = 1.0 \times 10^{-4} \text{ s}^{-1}$	Degradation rate of Pro-caspase-8 that binds to FADD	Estimated
$k_{39} = 1.7 \times 10^{-4} \text{ s}^{-1}$	Degradation rate of Pro-caspase-8 that binds to RIP3	Fitted
$k_{40} = 5.5 \times 10^{-5} \text{ s}^{-1}$	Degradation rate of Ppm1b that binds to RIP3	Fitted
$k_{41} = 1.8 \times 10^{-3} \text{ s}^{-1}$	Degradation rate of RIP3 that binds to RIP1	Fitted
$k_{42} = 8.0 \times 10^{-5} \text{ s}^{-1}$	Degradation rate of RIP1 that binds to RIP3	Fitted
$k_{43} = 1.0 \times 10^{-4} \text{ s}^{-1}$	Degradation rate of FADD that binds to TRADD	Estimated
$k_{44} = 1.0 \times 10^{-3} \text{ s}^{-1}$	Degradation rate of Pro-caspase-8 that binds to FADD	Estimated
$k_{45} = 6.0 \times 10^{-4} \text{ s}^{-1}$	Degradation rate of Caspase-8 that activated by TRADD	Estimated
$k_{46} = 3.0 \times 10^{-5} \text{ s}^{-1}$	Degradation rate of Caspase-3	Estimated

Parameters in Model 4

Parameters	Description	References
$k_1^{\text{on}} = 3.83 \times 10^{-7} \text{ mpc}^{-1} \cdot \text{s}^{-1}$, $k_1^{\text{off}} = 1.0 \times 10^{-3} \text{ s}^{-1}$	Rates of TNF binds to TNFR1	Fitted and (Albeck et al., 2008)
$k_2 = 4.1 \times 10^{-1} \text{ s}^{-1}$	Rate of TNFR1 drops from TNF_TNFR1 complex	Fitted
$k_3 = 4.78 \times 10^{-4} \text{ s}^{-1}$	Inactivation rate of the dropped TNFR1	Fitted
$k_4^{\text{on}} = 3.07 \times 10^{-9} \text{ mpc}^{-2} \cdot \text{s}^{-1}$, $k_4^{\text{off}} = 1.0 \times 10^{-3} \text{ s}^{-1}$	Rate of TNF_TNFR1 complex recruits TRADD	Fitted and (Albeck et al., 2008)
$k_5 = 2.29 \times 10^{-4} \text{ s}^{-1}$	TRADD disassociation rate from Complex I	Fitted
$k_6 = 1.0 \times 10^{-7} \text{ s}^{-1}$	Inactivation rate of the dropped TRADD	Estimated
$k_7 = 5.19 \times 10^{-4} \text{ s}^{-1}$	Rate of TNFR1 drops from TNF_TNFR_TRADD complex	Fitted
$k_8^{\text{on}} = 2.38 \times 10^{-10} \text{ mpc}^{-2} \cdot \text{s}^{-1}$, $k_8^{\text{off}} = 1.0 \times 10^{-3} \text{ s}^{-1}$	Rate of TNF_TNFR1 complex recruits RIP1	Fitted and (Albeck et al., 2008)
$k_9 = 9.69 \times 10^{-4} \text{ s}^{-1}$	RIP1 disassociation rate from Complex I	Fitted
$k_{10} = 1.0 \times 10^{-7} \text{ s}^{-1}$	Inactivation rate of the dropped RIP1	Estimated
$k_{11} = 5.33 \times 10^{-4} \text{ s}^{-1}$	Rate of TNFR1 drops from TNF_TNFR_RIP1 complex	Fitted
$k_{12}^{\text{on}} = 7.0 \times 10^{-10} \text{ mpc}^{-1} \cdot \text{s}^{-1}$, $k_{12}^{\text{off}} = 1.0 \times 10^{-3} \text{ s}^{-1}$	Rates of RIP1 binds to FADD	Fitted and (Albeck et al., 2008)
$k_{13} = 1.0 \text{ s}^{-1}$	Activation rate of FADD	(Albeck et al., 2008)
$k_{14} = 1.0 \times 10^{-8} \text{ mpc}^{-1} \cdot \text{s}^{-1}$	Rates of Pro-caspase-8 binds to FADD	Estimated
$k_{15} = 9.89 \times 10^{-4} \text{ s}^{-1}$, $n_{15} = 4$,<br>$K_{\text{pC8}} = 10^{-2} \text{ mpc}$, $K_{\text{C8R}} = 1.83 \times 10^3 \text{ mpc}$	Rate and michaelis constant of Caspase-8 activation	Estimated
$k_{16} = 8.0 \times 10^{-5} \text{ s}^{-1}$, $K_{\text{RIP1}} = 1.0 \text{ mpc}$, $n_{16} = 15$	Rate and michaelis constant of RIP3 binds to RIP1	Fitted
$k_{17} = 6.84 \times 10^{-5} \text{ s}^{-1}$, $K_{\text{RIP3}} = 16 \text{ mpc}$, $n_{17} = 2$	Rate and michaelis constant of RIP3 phosphorylation	Estimated
$k_{18}^{\text{on}} = 1.0 \times 10^{-2} \text{ mpc}^{-1} \cdot \text{s}^{-1}$, $k_{18}^{\text{off}} = 1.0 \times 10^{-2} \text{ s}^{-1}$	Rates of Pro-caspase-8 inhibits RIP3	(Albeck et al., 2008)
$k_{19}^{\text{on}} = 1.0 \times 10^{-2} \text{ mpc}^{-1} \cdot \text{s}^{-1}$, $k_{19}^{\text{off}} = 1.0 \times 10^{-2} \text{ s}^{-1}$	Rates of Caspase-8 inhibits p-RIP3	(Albeck et al., 2008)
$k_{20}^{\text{on}} = 1.0 \times 10^{-2} \text{ mpc}^{-1} \cdot \text{s}^{-1}$, $k_{20}^{\text{off}} = 1.0 \times 10^{-2} \text{ s}^{-1}$	Rates of Bar inhibits Caspase-8	(Albeck et al., 2008)
$k_{21} = 4.0 \times 10^{-2} \text{ mpc} \cdot \text{s}^{-1}$, $K_{\text{RIP3-1}} = 100 \text{ mpc}$	Rate and michaelis constant of RIP1 binds to RIP3	Fitted
$k_{22} = 1.4 \times 10^{-5} \text{ mpc} \cdot \text{s}^{-1}$, $K_{\text{Ppm1b}} = 10 \text{ mpc}$, $n_{22} = 4$	Rate and michaelis constant of Ppm1b binds to RIP3	Fitted
$k_{23}^{\text{on}} = 9.66 \times 10^{-4} \text{ mpc}^{-1} \cdot \text{s}^{-1}$, $K_{\text{Ppm1bbind}} = 10^{-2} \text{ mpc}$,<br>$n_{23} = 4$, $k_{23}^{\text{off}} = 3.53 \times 10^{-2} \text{ s}^{-1}$	Rates of negative effect of Ppm1b on RIP3	Estimated
$k_{24}^{\text{on}} = 4.5 \times 10^{-10} \text{ mpc}^{-1} \cdot \text{s}^{-1}$, $k_{24}^{\text{off}} = 1.0 \times 10^{-3} \text{ s}^{-1}$	Rates of MLKL binds to RIP3	Fitted and (Albeck et al., 2008)
$k_{25} = 1.0 \text{ s}^{-1}$	Activation rate of MLKL	(Albeck et al., 2008)

$k_{26}^{on} = 1.5 \times 10^{-7} \text{ mpc}^{-1} \cdot \text{s}^{-1}, k_{26}^{off} = 1.0 \times 10^{-4} \text{ s}^{-1}$	Phosphorylation rates of MLKL	Estimated
$k_{27} = 1.0 \times 10^{-5} \text{ s}^{-1}, K_{C8} = 1.0 \times 10^4 \text{ mpc}, n_{27} = 4$	Rate and michaelis constant of Caspase-3 activation	Estimated
$k_{28} = 1.0 \times 10^{-4} \text{ s}^{-1}, K_{FADDT} = 2.5 \times 10^3 \text{ mpc}, n_{28} = 20$	Rates of TRADD binds to FADD	Estimated
$k_{29} = 1.0 \times 10^{-6} \text{ mpc}^{-1} \cdot \text{s}^{-1}$	Rates of Pro-caspase-8 binds to FADD	Estimated
$k_{30} = 1.6 \times 10^{-3} \text{ s}^{-1}, K_{pC8T} = 10^{-2} \text{ mpc}, n_{30} = 4, K_{C8T} = 10 \text{ mpc}$	Rate and michaelis constant of Caspase-8 activation	Estimated
$k_{31} = 2.0 \times 10^{-4} \text{ s}^{-1}, K_{C8} = 1.0 \times 10^4 \text{ mpc}, n_{31} = 4$	Rate and michaelis constant of Caspase-3 activation	Estimated
$k_{34} = 1.0 \times 10^{-6} \text{ mpc}^{-1} \cdot \text{s}^{-1}$	Rates of Pro-caspase-8 inhibits RIP1	Fitted
$k_{36} = 1.0 \times 10^{-4} \text{ s}^{-1}$	Degradation rate of dropped RIP1	Fitted
$k_{37} = 8.0 \times 10^{-5} \text{ s}^{-1}$	Degradation rate of FADD that binds to RIP1	Fitted
$k_{38} = 1.0 \times 10^{-4} \text{ s}^{-1}$	Degradation rate of Pro-caspase-8 that binds to FADD	Estimated
$k_{39} = 1.7 \times 10^{-4} \text{ s}^{-1}$	Degradation rate of Pro-caspase-8 that binds to RIP3	Fitted
$k_{40} = 5.5 \times 10^{-5} \text{ s}^{-1}$	Degradation rate of Ppm1b that binds to RIP3	Fitted
$k_{41} = 1.8 \times 10^{-3} \text{ s}^{-1}$	Degradation rate of RIP3 that binds to RIP1	Fitted
$k_{42} = 8.0 \times 10^{-5} \text{ s}^{-1}$	Degradation rate of RIP1 that binds to RIP3	Fitted
$k_{43} = 1.0 \times 10^{-4} \text{ s}^{-1}$	Degradation rate of FADD that binds to TRADD	Estimated
$k_{44} = 1.0 \times 10^{-3} \text{ s}^{-1}$	Degradation rate of Pro-caspase-8 that binds to FADD	Estimated
$k_{45} = 6.0 \times 10^{-4} \text{ s}^{-1}$	Degradation rate of Caspase-8 that activated by TRADD	Estimated
$k_{46} = 3.0 \times 10^{-5} \text{ s}^{-1}$	Degradation rate of Caspase-3	Estimated
$k_a = 1.0, k_d = 5.0 \times 10^{-8}$	Rates of inherent cells growth and decay	Fitted
$k_{\text{Caspase-3}} = 8.0 \times 10^{-6}, k_{\text{MLKL}} = 5.14 \times 10^{-5}$	Rates of Caspase-3 and MLKL induce cell death	Fitted
$N = 2 \times 10^7$	Population of cells	Measured

Reference

J. G. Albeck, J. M. Burke, S. L. Spencer, D. A. Lauffenburger, P. K. Sorger, Modeling a Snap-Action, Variable-Delay Switch Controlling Extrinsic Cell Death. *Plos Biology* **6**, 2831-2852 (2008).
